# Field-to-forest fungal spillover is a biotic edge effect of coffee in Costa Rica

**DOI:** 10.1038/s41467-026-75178-3

**Published:** 2026-07-24

**Authors:** Jeffrey A. Lackmann, Bénédicte Bachelot, Catherine Lindell, Elena Prado-Ragan, Priscila Chaverri, Efraín Escudero-Leyva, Gina Errico, Megan Orr, Federico Oviedo Brenes, Jeisson Figueroa, Laura Aldrich-Wolfe

**Affiliations:** 1 North Dakota State University, Department of Biological Sciences, Fargo, ND USA; 2 University of Hawaii at Manoa, Pacific Biosciences Research Center, Honolulu, HI USA; 3 Oklahoma State University, Dept. of Biology, Stillwater, OK USA; 4 Michigan State University, Department of Integrative Biology and Center for Global Change and Earth Observations, East Lansing, MI USA; 5 Michigan State University, Ecology, Evolution, and Behavior Program, East Lansing, MI USA; 6 Bowie State University, Department of Natural Sciences, Bowie, MD USA; 7 Universidad de Costa Rica, Centro de Investigación en Productos Naturales (CIPRONA), San José, Costa Rica; 8 North Dakota State University, Department of Statistics, Fargo, ND USA; 9 Ecos del Bosque, Buena Vista, Costa Rica; 10 Organization for Tropical Studies, Las Cruces Biological Station, San Vito, Costa Rica

**Keywords:** Tropical ecology, Agroecology, Forest ecology, Fungal ecology

## Abstract

Agricultural expansion creates mosaic landscapes where managed land borders natural ecosystems, facilitating the movement of organisms between habitats. Ecological spillover from natural systems to crops has been previously examined, but the extent to which populations amplified by agriculture move into adjacent forests remains largely unexplored. We investigated whether Costa Rican coffee fields act as reservoirs for foliar fungi that, because of high crop density and phylogenetic signal in plant-fungal associations, may particularly affect close relatives of coffee (Rubiaceae) in the forest understory. Field surveys, airborne propagule sampling, and metabarcoding along transects at the coffee-forest border revealed declining leaf spot incidence with distance into the forest for Rubiaceae but not non-Rubiaceae hosts, in parallel with declines in the dominant fungal order on coffee leaves, Pleosporales, and 19 coffee-associated taxa. Ninety-three coffee-associated taxa, including many potential plant pathogens, showed higher modeled abundance on forest Rubiaceae than forest non-Rubiaceae hosts. Our findings suggest that crops can act as reservoirs for fungi colonizing adjacent forest plants, disproportionately affecting hosts more closely related to the crop. These plants may face increased disease pressure, leading to changes in forest understory composition near the crop-forest border. Phylogenetically structured spillover represents an underappreciated edge effect in fragmented landscapes.

## Abstract (web summary)

Agriculture can affect wild ecosystems when crop microbes spread to nearby plants. Here, coffee-associated fungi were found to disproportionately colonize wild relatives of coffee near forest–farm borders.

## Introduction

Foliar fungi inhabit leaf surfaces and interiors, exhibit high dispersal potential^1^, varying degrees of host specificity^2^, and distinct functional roles as both pathogens and mutualists capable of mediating plant community structure^3^. The widespread conversion of natural ecosystems to agriculture creates mosaic landscapes where managed land directly borders wildlands^4,5^. Edges between managed and natural communities create ecological interfaces where foliar fungi from both communities come into contact. These fungi and their associated functions in the ecosystem can move across these boundaries in a phenomenon known as ecological spillover^6,7^.

Ecotones between agricultural and natural systems are often characterized by pronounced edge effects, where environmental conditions, species composition, and biotic interactions differ markedly from the ecosystem interiors^8,9^. In tropical forest landscapes fragmented by agriculture, edges are known to alter microclimate, species turnover, and ecological processes^10^, including the spread of fungal pathogens^11^. High-density monocultures, such as coffee plantations, often generate stark biotic borders between managed and natural biotic communities, where augmented abundances of managed host plants and associated pathogens may alter disease pressure in adjacent natural communities^12^. While many studies have focused on natural systems as reservoirs of organisms that affect nearby agricultural systems, much less is known about the extent to which organisms from agriculture disperse into and modify adjacent natural systems^6,13^. Many foliar fungi associate closely with managed systems and may accumulate at high enough densities to spill into adjacent wildlands^14^. Spillover at agricultural-forest interfaces is inherently bidirectional: forests can serve as reservoirs of organisms that affect crops, while agricultural systems may likewise generate or amplify organisms that disperse into adjacent natural communities. However, this latter direction has received comparatively little research attention.

In forests, enemy pressure at higher conspecific densities limits dominant species and promotes the persistence of rare species, a phenomenon commonly referred to as Janzen-Connell effects^15,16^. Analogous density-dependent processes also drive disease outbreaks in managed crop systems when conditions favor host-associated pathogens^17,18^. High-density crop systems amplify populations of pathogens, pests, and natural enemies, increasing the potential for ecological spillover into adjacent natural systems. The likelihood of spillover also depends on host relatedness: foliar fungi are more likely to establish in nearby habitats when they encounter compatible hosts^19^. While some fungi (e.g., *Xylaria* spp.) have broad host ranges, others (e.g., *Colletotrichum* spp.) specialize on particular plant lineages^20–23^. Thus, in natural systems, plants closely related to a crop species should be more susceptible to colonization by crop-associated fungi than more distantly related neighbors^24,25^. If these fungi influence host fitness, they could alter plant community composition over time, favoring close relatives of the crop if they act as mutualists or selecting against them if pathogenic^26^.

**Fig. 1:**
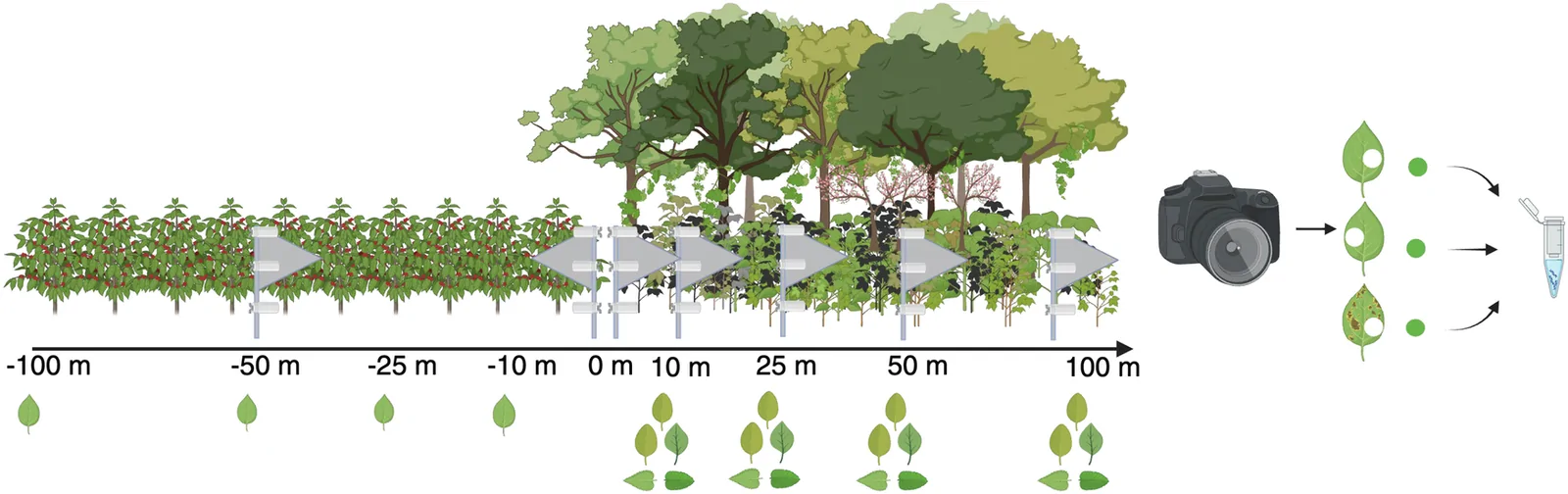
Sampling foliar and airborne fungi across Costa Rican coffee-forest borders. Coffee-forest transects were established at eight sites. Rotating passive air samplers were deployed to capture airborne fungal propagules at 50 m from the coffee-forest border in the coffee and at 10, 25, 50, and 100 m into the forest, and fixed-position passive air samplers were located at the border, one facing coffee and the other forest, to sample propagules moving from one habitat to the other. Single leaves were collected from 4 to 5 coffee plants at 10, 25, 50 m for all sites and 100 m into coffee at seven sites (at one site, RN, the transect extended only 50 m into coffee due to field size). Leaves were sampled from 2 to 6 individuals of three plant species in the Rubiaceae family and two plant species in non-Rubiaceae families at 10, 25, 50 and 100 m into the forest. If sufficient individuals could not be located, additional species were sampled within each host type. Leaves were photographed, screened for pathogen damage, and a sample was removed for DNA extraction. Created in BioRender. Lackmann, J. (2026) https://BioRender.com/zh6o256.

The foliar fungal communities of coffee plantations in Central America provide an ecologically and economically relevant tropical system for assessing whether ecological spillover of crop-associated fungi occurs and whether nearby, close relatives of the host plant are more likely to be colonized than more distantly related plant species. Coffee (*Coffea arabica* L., Family Rubiaceae), as one of the most economically important crops in the tropics, plays a central role in shaping both rural livelihoods and land use^27^. These plantations are often managed as high-density monocultures in close proximity to remnant forest^28^. The Rubiaceae family includes many native forest understory species in Central America^29,30^. Thus, the high density of the crop, its physical proximity to forests, and the phylogenetic overlap with many forest plants create conditions where ecological spillover of coffee-associated phyllosphere fungi from field to forest is likely. In this study, we consider whether coffee fields act as reservoirs for foliar fungi that can affect adjacent forest understory plants, particularly those belonging to the coffee family (Rubiaceae), by characterizing visible disease symptoms in conjunction with foliar and airborne fungal communities of coffee and adjacent understory forest plants along transects spanning coffee-forest borders (Fig. 1). We hypothesize that if coffee serves as a reservoir, we would detect foliar fungi at higher abundances in the air and in the leaves of plants in the forest near the coffee-forest border compared to plants located further into the forest, with a concomitant pattern in disease symptoms. Second, we hypothesize that if host relatedness affects colonization, coffee-associated fungi should occur more frequently on forest plants the more closely related they are to coffee.

## Results

### Leaf spot on coffee and forest understory leaves and seedling fungal damage

**Fig. 2:**
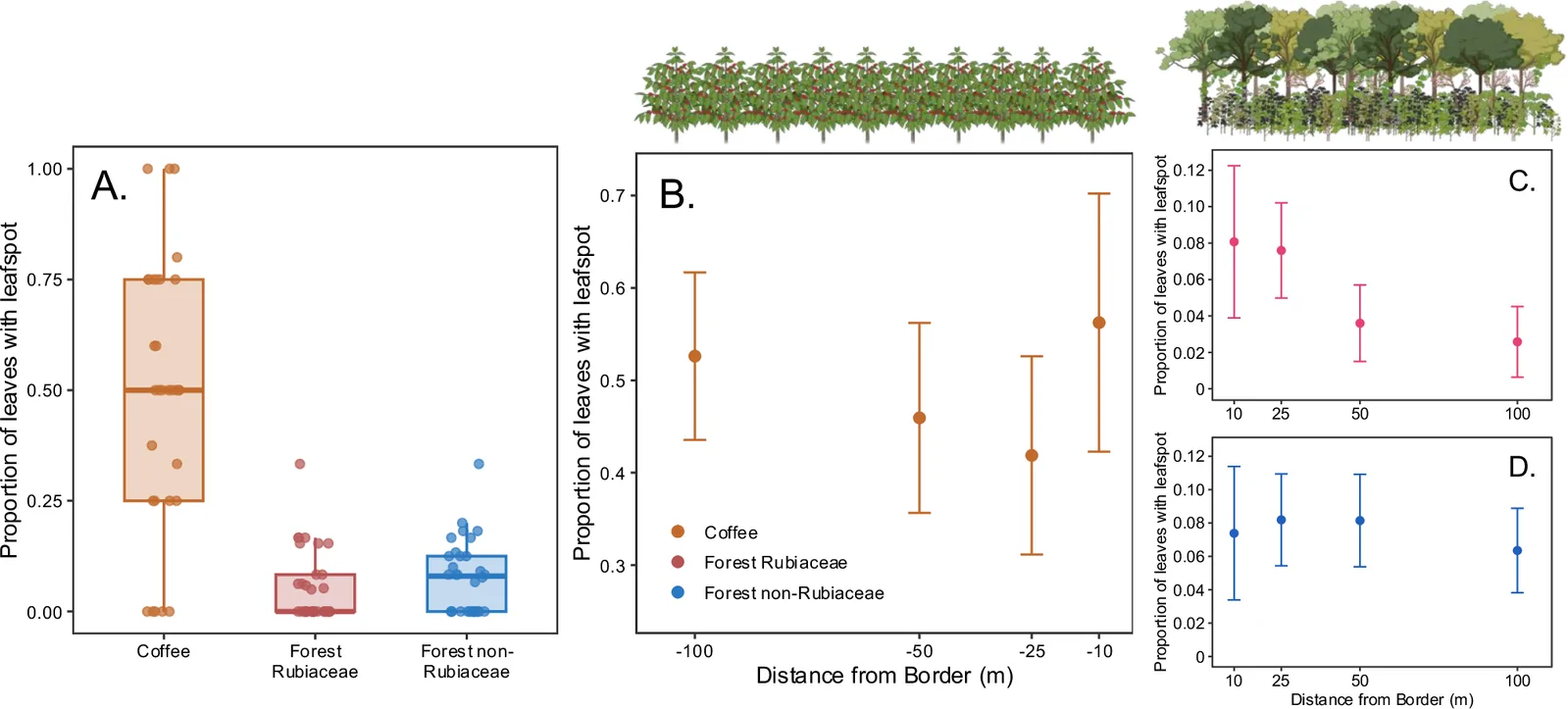
Foliar fungal disease symptoms prevalent in coffee declined with distance into adjacent forest on close coffee relatives. The proportion of leaves sampled with leaf spot for coffee and forest understory plants at all distances at eight farms (**A**; *n* = 31 for coffee; *n* = 32 each for forest Rubiaceae and forest non-Rubiaceae); and at each sampling distance for *Coffea arabica* (**B**; *n* = 8, except *n* = 7 at 100 m), **C** understory forest plants in the coffee family, Rubiaceae, (*n* = 8) and, **D** forest understory plants in non-Rubiaceae families (*n* = 8). For **B–D** Values are means ± SE. *Y*-axis scales differ for coffee vs. forest data. According to binomial generalized linear mixed models coffee leaves were significantly more likely to exhibit leaf spot than forest leaves (Wald *χ*²(_1, 934_) = 15.07, *p* = 0.0001, *β*_forest_ = −2.434, 95% *CI* = [ − 3.66, −1.21]. Leaf spot incidence declined with distance into the forest for forest Rubiaceae (Wald *χ*²(_1,446)_ = 4.36, *p* = 0.0367, *β*_distance_ = −0.579, 95% *CI* = [ − 1.12, −0.04]) but not non-Rubiaceae (Wald *χ*²(_1,349_) = 0.08, *p* = 0.7826, *β*_distance_ = −0.059, 95% *CI* = [ − 0.48, 0.36]. For boxplots in (**A**), center lines indicate medians, box limits indicate the 25th (Q1) and 75th (Q3) percentiles, and box height represents the interquartile range (IQR = Q3 − Q1). Whiskers extend to the most extreme values within 1.5 × IQR of Q1 and Q3, and points beyond whiskers are plotted as outliers. The number of leaves sampled for each host type at each distance is provided in Table 1. Components of this figure were created in BioRender. Lackmann, J. (2026) https://BioRender.com/ri5lgic.

We found that the odds of leaf spot incidence were lower on forest understory plants than on coffee (Fig. 2A, B). In forest Rubiaceae, the odds of leaf spot incidence declined with distance into the forest from the coffee–forest border (Fig. 2C). However, for non-Rubiaceae plants in the forest understory, there was no effect of distance from the border on leaf spot incidence (Wald *χ*²(_1,349_) = 0.08, *p* = 0.7826, *β*_distance_ = −0.059, 95% *CI* = [ − 0.48, 0.36]; Fig. 2D). There was no effect of phylogenetic distance from coffee on incidence of leaf spot in forest Rubiaceae or non-Rubiaceae (Wald χ²(_1,446_) = 0.19, *p* = 0.6652, *β*_phylodist_ = −0.12, 95% CI [ − 0.68, 0.44].; Wald χ²(_1,349_) = 2.38, *p* = 0.1226, *β*_phylodist_ = 0.51, 95% CI [ − 0.14, 1.15]; Table S8) and no interaction between distance and phylogenetic distance for either Rubiaceae or non-Rubiaceae (Wald χ²(_1,446_) = 0.52, *p* = 0.4724, *β*_distance*phylodist_ = 0.18, 95% CI [ − 0.31, 0.67]; Wald χ²(_1,349_) = 0.93, *p* = 0.3355, *β*_distance*phylodist_ = −0.22, 95% CI [ − 0.66, 0.23]; Table S8).

**Fig. 3:**
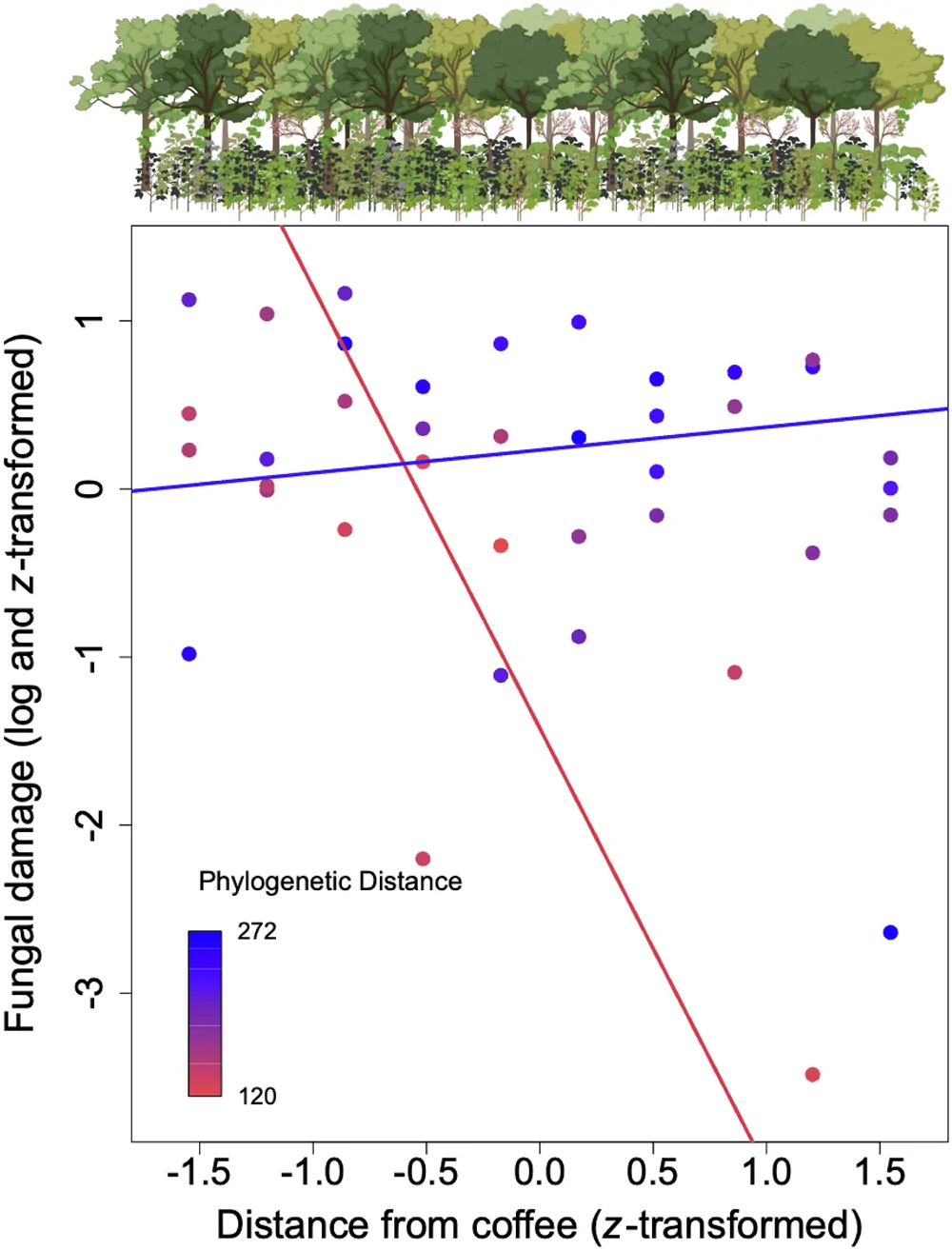
Pathogen damage of seedlings disproportionately affects close relatives of coffee near the coffee-forest border. Relationship between distance from the coffee–forest border and pathogen damage on forest seedlings surveyed in 1 m² plots spaced every 10 m from 5–105 m inside the forest at four of the eight sites for a total of 40 plots (*n* = 4 plots at each distance). Dots are colored on a continuous scale from red to blue based on the average phylogenetic distance to coffee of seedlings in each plot beginning with the minimum distance and ending with the maximum. Solid lines show model-predicted relationships from a linear mixed-effects model with site as a random effect, plotted at the minimum and maximum observed values of plot-level phylogenetic distance after log(1 + *x*) transformation and *z*-standardization. Pathogen damage was quantified as the mean percentage of leaf area affected per plot. Damage declined with distance from the border in close relatives of coffee, but this decline weakened as phylogenetic distance increased (*t*(_35_) = 2.56, *p* = 0.0148, *β*_distance*phylodist_ = 0.52, 95% CI [0.12, 0.91]). Axes show standardized variables: *x* = *z-*standardized distance from the coffee border and *y* = *z-*standardized log-transformed mean fungal damage of seedlings in each plot (log(1 + damage)). Negative values indicate observations below the overall mean. Components of this figure were created in BioRender. Lackmann, J. (2026) https://BioRender.com/ri5lgic.

For seedlings surveyed for fungal damage, there was a positive interaction between phylogenetic distance from coffee and physical distance from the coffee field in predicting fungal damage at the plot level (*t*(_35_) = 2.56, *p* = 0.0148, *β*_distance*phylodist_ = 0.52, 95% CI [0.12, 0.91]; Fig. 3), indicating that the negative effect on disease levels of physical distance is strongest in hosts closely related to coffee and is weaker in distantly related hosts.

### Airborne fungi from coffee to forest

**Fig. 4:**
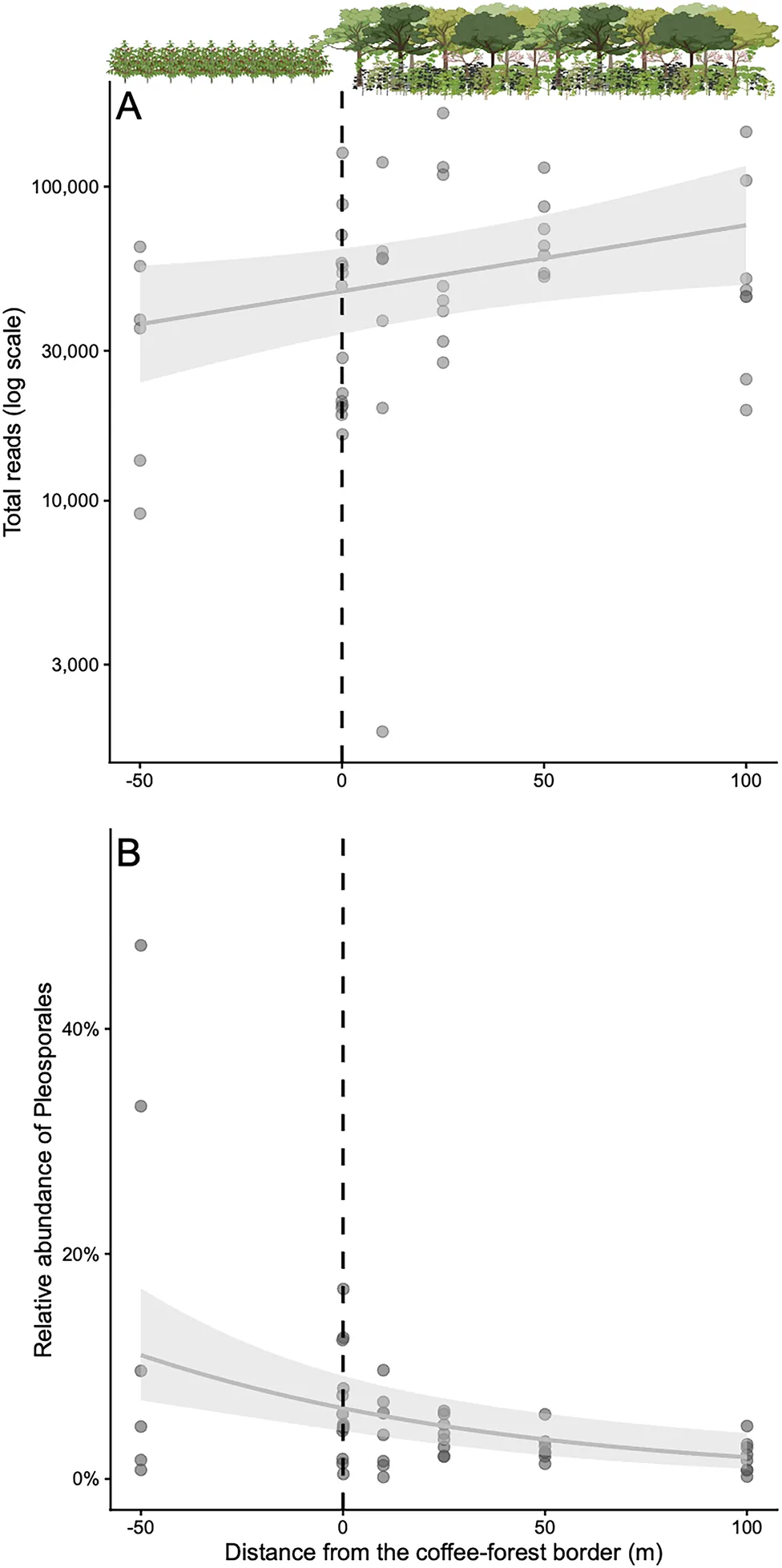
The relative abundance of airborne Pleosporales declined from coffee field to forest interior. **A** The total reads (log scale) of all airborne fungi and **B** relative abundance of Pleosporales at each sampling distance along the coffee-to-forest transects across four months (April-July, *n* = 8 samples from each distance except *n* = 7 at −100). Negative values indicate distances into the coffee fields. *Y*-axes differ. Total read count increased, while the relative abundance of Pleosporales declined, with distance into the forest (Wald χ²(_1,52_) = 5.12, *p* = 0.0236, *β*_distance_ = 0.005, 95% CI [0.001, 0.009]; Wald χ²(_1,52_) = 14.83, *p* = 0.0001, *β*_distance_ = −0.01, 95% CI [ − 0.02, −0.01], respectively). Lines show fixed-effect predictions of our generalized linear mixed models (**A** = negative binomial family; **B** = beta binomial family) and shaded bands indicate approximate 95% confidence intervals on the response scale after back-transformation. Components of this figure were created in BioRender. Lackmann, J. (2026) https://BioRender.com/t0qbym.

From the air samples we collected, the abundance table contained 2,465,390 reads and 6838 OTUs. Total fungal read counts in the air increased with distance along the transect into the forest (Wald χ²(_1,52_) = 5.12, *p* = 0.0236, *β*_distance_ = 0.005, 95% CI [0.001, 0.009]; Fig. 4A). At the coffee-forest border, there was no difference in read counts between air samplers facing the forest or coffee (Wald χ²(_1,10_) = 0.6989, *p* = 0.4032, *β*_distance_ = 1.2358, 95% CI = [−1.6615, 4.1332]). In contrast, the relative abundance of airborne Pleosporales (542 OTUs) declined with increasing distance into the forest (Wald χ²(_1,52_) = 14.83, *p* = 0.0001, *β*_distance_ = −0.01, 95% CI [ − 0.02, −0.01]; Fig. 4B). This pattern remained significant when the analysis was restricted to forest-interior samplers, indicating that the decline was not driven solely by elevated abundance at the forest edge (Table S8).

**Fig. 5:**
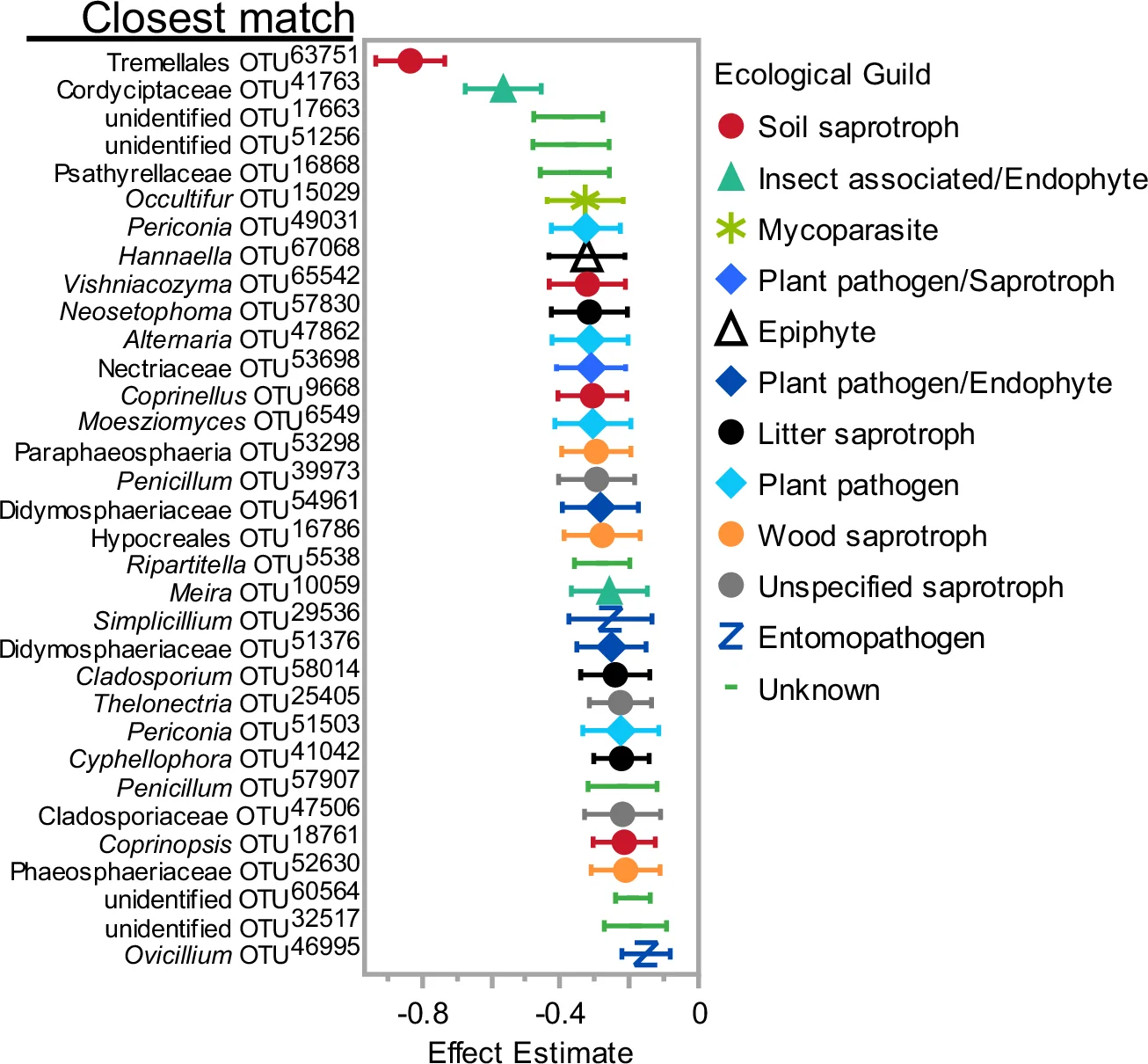
Coffee-associated airborne fungi that exhibited declines with distance into the forest. Values are mean effect estimates + SE from our generalized joint attribute model (GJAM)^72^ (Supplementary Materials Table S5). Airborne samples were collected monthly for four months (for six of the eight sites; two sites were sampled monthly for three and two months, respectively) using passive air samplers at 0 (forest-facing), 10, 25, 50, and 100 m into the forest interior (*n* = 29 airborne samples at each distance across all sites). More negative effect estimates indicate stronger negative associations with distance on the GJAM latent scale, but do not represent direct fold-changes or proportional declines in relative abundance. Ecological guilds were assigned using FungalTraits^74^ in conjunction with manual screening.

Among the fungal OTUs detected in the air, 33 were negatively associated with distance from the coffee-forest border into the forest interior (Fig. 5 and Supplementary Materials Table S5). These taxa included saprotrophs (13 OTUs), and potential plant pathogens/endophytes (9 OTUs), two entomopathogens, one epiphyte and one mycoparasite. Seven of the thirty-three could not be assigned to an ecological guild.

### Foliar fungi from the coffee-forest border into the forest interior

**Fig. 6:**
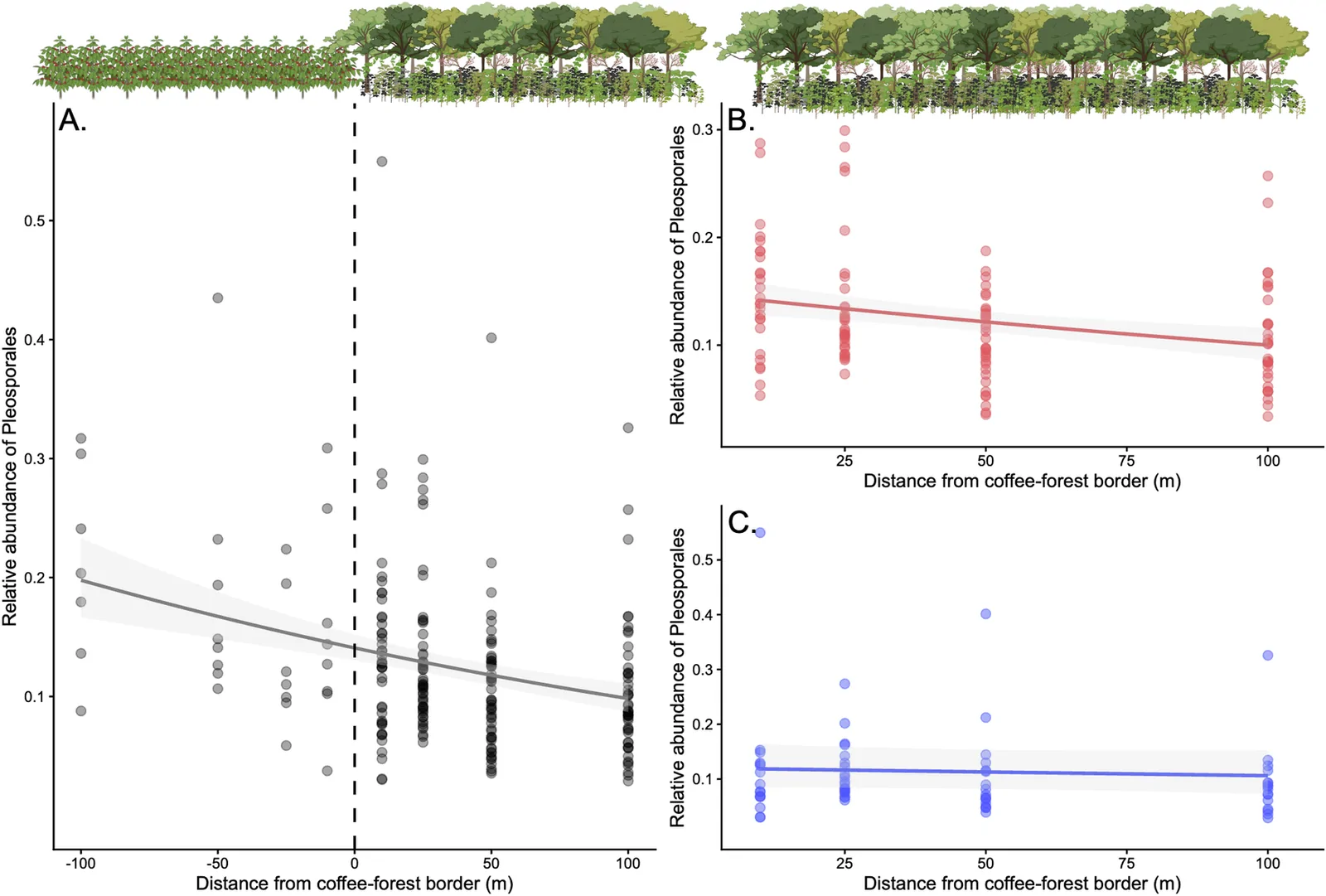
The relative abundance of Pleosporales on leaves declined from coffee field to forest interior only on close relatives of coffee. The relative abundance of Pleosporales at each sampling distance along coffee-forest transects plotted with the overlayed output from our beta-binomial generalized linear mixed models for **A** all sampled leaves (negative values denote distance into coffee from coffee-forest edge) **B** forest Rubiaceae leaves and **C** forest non-Rubiaceae leaves. Lines show fixed-effect predictions (from generalized linear mixed models: beta-binomial family) and shaded bands indicate approximate 95% confidence intervals on the response scale after back-transformation. The relative abundance of Pleosporales decreased with distance into the forest from the coffee-forest border in our model of all molecular leaf samples (Wald χ²(_1,217_) = 27.90, *p* = 1.28 × 10⁻⁷, *β*_distance_ = −0.0041, 95% CI [ − 0.0056, −0.0026]) and with distance from the coffee-forest border in forest Rubiaceae leaves (Wald χ²(_1,118_) = 11.45, *p* = 0.0007, *β*_distance_ = −0.0044, 95% CI [ − 0.0069, −0.0018]; No such spatial relationship was observed in non-Rubiaceae forest leaves (Wald χ²(_1,65_) = 0.47, *p* = 0.4928, *β*_distance_ = −0.0014, 95% CI [ − 0.0054, 0.0026]). Points indicate the relative abundance of Pleosporales in each host species at each distance at each site. The number of molecular leaf samples is summarized in Table 1. Components of this figure were created in BioRender. Lackmann, J. (2026) https://BioRender.com/tsw78s9.

The OTU abundance table for foliar fungal communities contained 5,970,846 reads and 9905 OTUs. The relative abundance of Pleosporales (932 OTUs) declined with physical distance into the forest on Rubiaceae but not non-Rubiaceae hosts (Wald χ²(_1,118_) = 11.45, *p* = 0.0007, *β*_distance_ = −0.0044, 95% CI [ − 0.0069, −0.0018]; Wald χ²(_1,65_) = 0.47, *p* = 0.4928, *β*_distance_ = −0.0014, 95% CI [ − 0.0054, 0.0026]; Fig. 6).

**Fig. 7:**
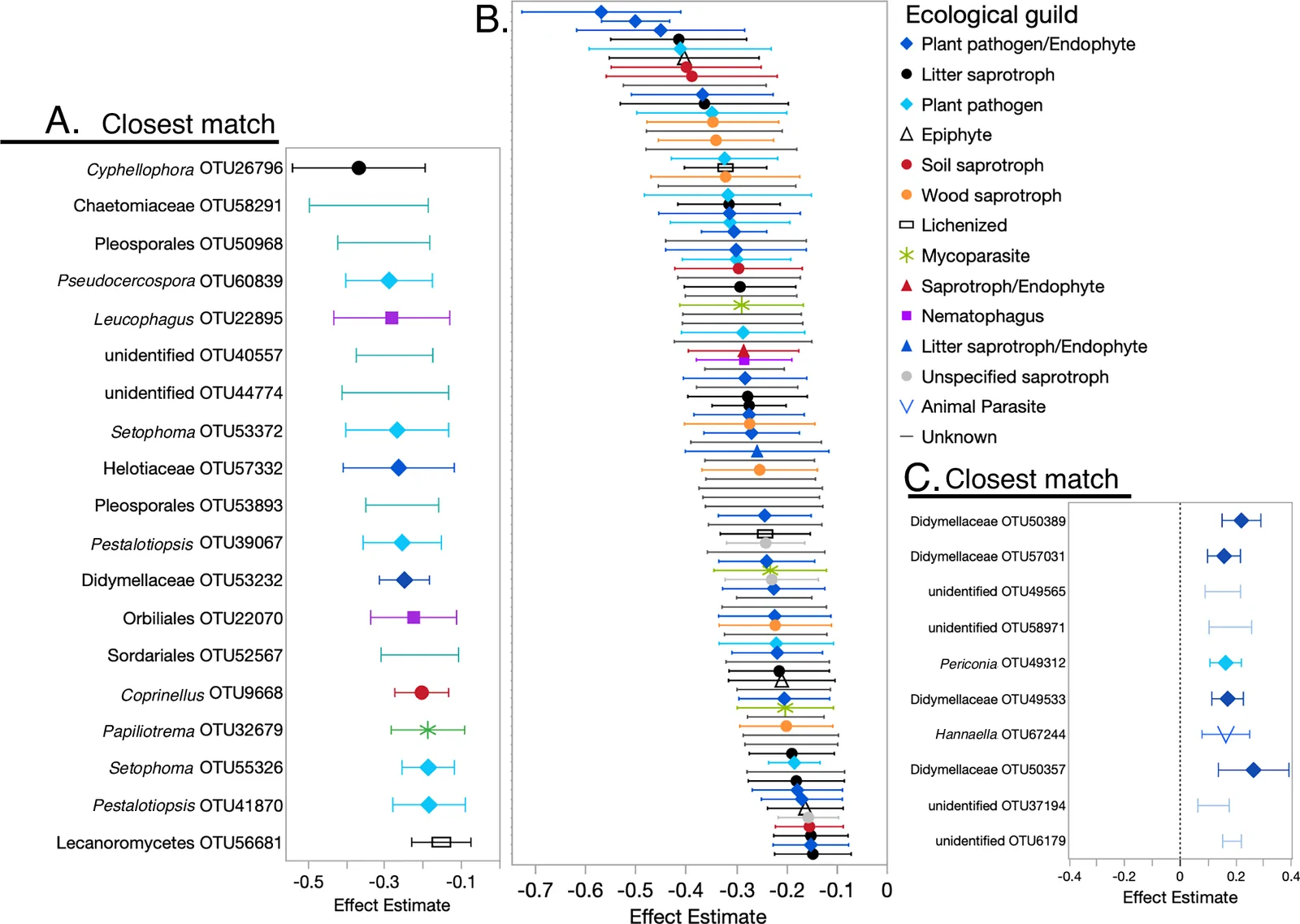
Coffee-associated fungi were negatively associated with both physical and phylogenetic distance of the host plant from coffee. All fungal OTUs from leaves that declined in abundance with **A** distance from the coffee border into the forest, **B** phylogenetic distance from coffee (Supplementary Materials Table S6, 7), and **C** an interaction of these two fixed effects according to our generalized joint attribute model (GJAM)^72^. Values are mean effect estimates ± SE and *n* = 42, 50, 52, and 47 molecular leaf samples at 10, 25, 50, and 100 m, respectively. More negative effect estimates indicate stronger negative associations with the predictor on the GJAM latent scale, but they do not represent direct fold changes or proportional declines in relative abundance. Ecological guilds were assigned using FungalTraits^74^ in conjunction with manual screening of existing literature.

Of the 1,000 most abundant OTUs in coffee (hereafter described as coffee-associated taxa), 836 were also observed on > 10 samples of forest understory leaves, making up the majority (52.4%) of the total reads in the entire foliar dataset. Of these 836 OTUs, 19 ( < 3%) were significantly negatively associated with distance into the forest (Fig. 7A and Supplementary Materials Table S6). These included several taxa whose closest matches were potential plant pathogens (seven OTUs), which may also function as endophytes depending on host context, as well as closest matches to nematophagous (two OTUs), saprotrophic (two OTUs), a mycoparasitic (one OTU), and lichenized (one OTU) taxa. Ninety-three OTUs were significantly positively associated with hosts more closely related to coffee than with those more distantly related (Fig. 7B). Among these taxa, nearly half of those assigned to an ecological guild were potential plant pathogens and endophytes (33 OTUs). The remaining taxa for which it was possible to assign a putative ecological guild included saprotrophs (25 OTUs), epiphytes and lichenized fungi (6 OTUs), mycoparasites (3 OTUs), and one nematophagous OTU (Fig. 7B and Supplementary Materials Table S7). An additional 33 OTUs could not be assigned to a guild. For 10 OTUs, there was a significant interaction between physical distance from the coffee edge and host phylogenetic distance. Because this pattern arises from an interaction between phylogenetic and physical distance, it can be equivalently described as either a weakening of the effect of host relatedness with increasing distance from coffee or as a spatial effect that is strongest on hosts closely related to coffee. In practice, this manifests as little to no effect of distance on distantly related hosts, consistent with phylogenetically structured spillover (Fig. 7C).

### Alpha and beta diversity of coffee, forest Rubiaceae, and forest non-Rubiaceae

Coverage-standardized alpha diversity differed consistently among vegetation groups, with coffee leaves supporting the lowest fungal diversity, forest non-Rubiaceae intermediate diversity, and forest Rubiaceae the highest diversity. This pattern was evident for both richness (Fig. S4A and Hill *q* = 0; Kruskal–Wallis χ²(2) = 64.29, *p* = 1.10 × 10⁻¹⁴, *ε*² = 0.29) and diversity (Fig. S4B and Hill *q* = 1; χ²(2) = 42.99, *p* = 4.62 × 10⁻¹⁰, *ε*² = 0.19). All pairwise contrasts were significantly different for richness (Wilcoxon rank-sum test with Benjamini-Hochberg correction: coffee vs forest Rubiaceae: *W* = 198, *p*_adj_ = 1.28 × 10⁻¹³; Coffee vs Forest non-Rubiaceae: *W* = 284, *p*_adj_ = 1.65 × 10⁻⁸; forest Rubiaceae vs forest non-Rubiaceae: *W* = 5489, *p*_adj_ = 0.0005) and diversity (coffee vs forest Rubiaceae: *W* = 488, *p*_adj_ = 1.59 × 10⁻⁹; coffee vs forest non-Rubiaceae: *W* = 468, *p*_adj_ = 2.41 × 10⁻⁵; forest Rubiaceae vs forest non-Rubiaceae: *W* = 5332, *p*_*adj*_ = 0.002).

Leaf fungal communities differed compositionally by host type (coffee, forest Rubiaceae, forest non-Rubiaceae) based on Bray–Curtis dissimilarities (PERMANOVA, 9999 permutations; pseudo-*F*(_2,218_) = 5.25, *p* = 0.0010, *R*² = 0.046), with significant pairwise differences in composition after Benjamini-Hochberg correction between coffee and forest Rubiaceae (pseudo-*F*(_1,150_) = 7.56, *p*_*adj*_ = 0.0001, *R*² = 0.048), coffee and forest non-Rubiaceae (pseudo-*F*(_1,97_) = 6.32, *p*_*adj*_ = 0.0001, *R*² = 0.061), and forest Rubiaceae and forest non-Rubiaceae (pseudo-*F*(_1,189_) = 2.91, *p*_*adj*_ = 0.0001, *R*² = 0.0152). Within forest samples, composition varied significantly overall (pseudo-*F*(_3,187_) = 1.76, *p* = 0.0001, *R*² = 0.0275), driven by host type (pseudo-*F*(_1,187_) = 2.92, *p*_*adj*_ = 0.0001, *R*² = 0.0152), but not continuous distance into the forest (pseudo-*F*(_1,187_) = 1.21, *p*_*adj*_ = 0.0820, *R*² = 0.0063) or the distance × host type interaction (pseudo-*F*(_1,187_) = 1.16, *p* = 0.1320, *R*² = 0.0061). Fungal communities of forest non-Rubiaceae communities were more heterogeneous than forest Rubiaceae communities (PERMDISP; *F*(_1,189_) = 29.57, *p* = 0.0001; as expected for more phylogenetically distant taxa; mean distance to centroid: 0.631 for forest non-Rubiaceae vs. 0.603 for forest Rubiaceae). There was no effect of distance on heterogeneity (PERMDISP *F*(_3,187_) = 0.11, *p* = 0.9673).

## Discussion

While ecological spillover can take place in either direction, from natural to managed systems or vice versa, previous work on ecological spillover has focused on natural ecosystems as reservoirs of organisms that influence adjacent agricultural systems, such as pollinators^31^, pests^32^, or pathogens^6^. Here, we demonstrate across multiple lines of inquiry and data types that a crop can influence the composition of foliar fungal communities in adjacent forest, amplify the relative abundance of pathogens, and increase disease pressure near the habitat boundary. This represents a biotic edge effect distinct from more commonly documented effects, such as changes in light, humidity, temperature, canopy cover, or plant density^33,34^. Over time, crop-driven pathogen spillover could selectively disadvantage plant taxa most closely related to the crop species. In this way, agricultural landscapes like those of this Costa Rican study may not merely fragment habitats but also restructure adjacent plant and fungal communities.

While the forest understory harbors a much more diverse phyllosphere than coffee (Supplementary Materials and Fig. S4), several patterns point to field-to-forest spillover of crop-associated fungi. High-density resource patches have been shown to elevate consumer pressure in adjacent habitats in other systems, such as seed predation on wild sunflowers near cultivated fields^35^ and grasshoppers' herbivory extending across habitat patches^36^. Consistent with these dynamics, we observed strikingly higher incidence of fungal disease in coffee, coupled with a decline in disease incidence with distance into the forest restricted to forest Rubiaceae. Non-Rubiaceae hosts showed no such response (Figs. 2, 3). If disease were driven only by microclimatic edge effects, we would expect a similar spatial response across host lineages. That the distance-decay of disease only occurred on hosts closely related to coffee aligns with well-established theory and evidence that disease pressure is jointly structured by host abundance and phylogenetic relatedness, whereby dense host populations act as reservoirs that disproportionately affect closely related species^24^.

Tellingly, we see the identical pattern in the relative abundance of Pleosporales, the most abundant and diverse order of fungi on coffee leaves, declining with distance from coffee both in the air (Fig. 4B) and on forest Rubiaceae leaves (Fig. 6B). This order is one of the most widespread foliar fungal lineages in tropical systems and a group that includes numerous plant pathogens responsible for leaf spot diseases^37^. Given their prolific airborne dispersal and capacity to colonize leaf surfaces^38–40^, the parallel decline of Pleosporales in air and leaf communities points to a gradient in propagule supply rather than environmental filtering. Notably, our border samplers facing coffee and forest did not show differences in total airborne propagule load, despite the forest phyllosphere's greater diversity. We interpret this to suggest that neither side of the interface is denser with fungal propagules overall, but that coffee disproportionately contributes particular taxa to the local airborne pool, as we observed with Pleosporales.

More than 100 coffee-associated fungal taxa showed significant positive associations with decreasing phylogenetic distance between forest hosts and coffee (Fig. 7B, C), consistent with the phylogenetic structuring of pathogen host ranges^19,41^. Over 25% of these taxa had pathogenic potential, including taxa assigned to *Colletotrichum*, *Setophoma*, *Cercospora*, and members of the Didymellaceae family, groups that include agents of coffee leaf spot epidemics in Costa Rican and other Neotropical coffee systems^42–44^. Together with the well-documented role of localized dispersal in structuring phyllosphere communities^45,46^, we contend these patterns are better explained by spillover from coffee onto compatible forest hosts than microclimatic edge effects alone^12^.

Broadly, field-to-forest spillover could disrupt the density-dependent processes that structure forest understory communities near agro-forest borders. Cross-habitat spillover from high-density resource patches has been recognized as a mechanism that elevates consumer pressure in adjacent communities^6,47^. Such dynamics do not require that these pathogens originate in coffee itself. Rather, many organisms we detected are likely already circulating among wild hosts but become amplified in the dense crop environment, increasing propagule pressure back onto nearby compatible forest plants, consistent with the idea that reservoir hosts elevate pathogen transmission into adjacent communities^48^. In this way, coffee fields may function both as primary sources of diseases, such as Coffee Leaf Rust (*Hemileia vastatrix*)^49^ and as amplifying reservoirs that generate feedbacks intensifying disease pressure at forest edges, akin to spillback in parasite ecology^50^. Conservation ecologists could explore how leveraging existing management strategies, such as buffer vegetation^51^ composed of species distantly related to the crop (e.g., forage grasses between coffee fields and forest), could mitigate crop-associated pathogen spillover into adjacent natural areas.

While the spatial distribution of fungal taxa was consistent with spillover from coffee, this work cannot directly trace the origin of fungal propagules found on or in forest plant tissues. Definitively confirming that forest-colonizing foliar fungi originate in coffee fields will require approaches that directly track fungal movement across habitat boundaries, including directional spore trapping, strain-resolved genomic or metagenomic analyses, or isotopic labeling. Another important follow-up would be to characterize fungal communities on wild Rubiaceae in forests, with or without adjacent coffee, compared with fungal communities on coffee in the region.

High-density crop systems, such as coffee fields, can influence fungal communities in adjacent forests through ecological spillover, disproportionately affecting forest understory plants that are closely related to the crop plant. Our findings suggest that coffee fields serve as reservoirs for coffee-associated plant pathogens more likely to colonize nearby Rubiaceae plants in the forest understory. This illustrates that ecological spillover need not arise solely from diversity in the source pool but can also emerge from a high-density host that amplifies propagule pressure on nearby environments. In the mosaic landscapes of Costa Rica, where agricultural land is in direct proximity to both forest fragments and protected forest areas, crop-mediated spillover of coffee pathogens into the forest could alter forest plant community composition by amplifying disease pressure on native Rubiaceae. This presents an underappreciated biotic edge effect that can alter the plant community composition of apparently intact natural ecosystems that may extend to other agricultural systems and geographic regions. It is therefore crucial to promote larger, more contiguous conservation areas, particularly in the highly biodiverse tropics where agricultural encroachment is widespread and increasing.

## Methods

### Study sites

Fieldwork was conducted from March to August 2021 at eight sites in Coto Brus, Puntarenas Province, Costa Rica, near the Las Cruces Biological Station (8°47′7″N, 82°57′32″W), in a tropical premontane ecosystem at an elevational range of 800–1133 m asl with mean annual rainfall of 4000 mm and mean annual temperature of 21 °C^52,53^. Each site contained a privately owned coffee field directly adjacent to old-growth tropical forest > 10 ha in size, classified as premontane wet forest transition to rain forest (forest type according to Holdridge Life Zone Classification System^54^, Supplementary Materials Table S1). Coffee fields ranged in age from 10 to 67 years and were managed conventionally, including application of fungicides and inorganic fertilizers. For information regarding understory plant composition at each site see Supplementary Materials (Fig. S3 and Table S2). At seven of the sites, we established a 200 m transect extending 100 m into both the coffee field and the adjacent forest ensuring that the 100 m mark was at least 100 m from any other edge of the habitat (Fig. 1). At the eighth site (RN), the transect extended only 50 m into the coffee to avoid sampling within 50 m of the opposite edge of the field. The sampling at these sites and subsequent DNA analysis were approved by the Comisión Nacional para la Gestión de la Biodiversidad (resolution No. R-004-2021-OT-CONAGEBIO).

### Leaf collection for foliar fungi

To assess the spatial patterns of foliar fungi in coffee fields and the adjacent forest understory, using flame-sterilized scissors we removed a leaf from the second fully expanded leaf pair (counting from the branch tip) from four randomly selected coffee plants within a 5 m radius at 10, 25, 50, and 100 m away from the coffee-forest border at seven sites, while doubling the samples at 50 m in the eighth site, to keep the number of coffee leaf samples consistent across the sites (Fig. 1). In order to minimize potential sampling biases, we randomly selected the branch from an individual plant to be sampled using a random number generator. The first number selected determined the side of the plant we sampled from and the second determined which branch we chose to remove a leaf from. We repeated this sampling process for understory forest plants ( > 50 cm in height with leaves within arm's reach) at 10, 25, 50, and 100 m into the forest, sampling in a 5 m radius from the distance point along the transect. To investigate whether forest plants shared more foliar fungi with coffee when closely related than more distantly related, we sampled leaves from 2 to 6 individuals each of five plant species (three Rubiaceae, two non-Rubiaceae) at each distance (Fig. 1 and Table 1). If at least three individuals of a given species could not be located at a given distance, additional species within the relevant host types (Rubiaceae or non-Rubiaceae) were sampled. We sampled the same forest plant species across distances and sites whenever possible, but because plant species diversity was so high, we often had to sample different plant species at different distances and sites (Supplementary Materials Table S3). In total, we sampled 131 coffee leaves (one leaf sample was lost prior to image analysis) and 807 forest leaves (Table 1) spanning 26 species and eight families (Supplementary Materials Table S3) from June 1-July 7th, 2021. Representative material from each forest plant species was collected, identified, and deposited in the herbaria at Las Cruces Biological Station and the National Herbarium of Costa Rica.

**Table 1:** Sampling effort for coffee and adjacent forest leaves at eight coffee farms in Costa Rica

		Number of leaf samples by distance by site								
Sample type	Distance from coffee-forest border (m)	AJ	AN	DV	EP	LZ	RB	RN	VP	Total
Coffee	-100	4(1)	4(1)	5(1)	4(1)	4(1)	3(1)	-	4(1)	28(7)
	-50	4(1)	4(1)	5(1)	4(1)	4(1)	4(1)	8(1)	4(1)	37(8)
	-25	4(0)	4(1)	5(1)	4(1)	4(1)	4(1)	4(1)	4(1)	33(7)
	-10	4(1)	4(1)	5(1)	4(1)	4(1)	4(1)	4(1)	4(1)	33(8)
	Total	16(3)	16(4)	20(4)	16(4)	16(4)	15(4)	16(3)	16(4)	131(30)
Forest Rubiaceae	10	12(4)	13(3)	10(2)	15(3)	14(4)	12(3)	8(2)	16(5)	100(26)
	25	12(4)	13(4)	15(3)	12(3)	13(4)	20(5)	12(3)	16(5)	113(31)
	50	17(4)	12(3)	20(6)	12(3)	11(4)	16(4)	13(4)	17(6)	118(34)
	100	12(4)	12(3)	19(5)	13(4)	16(4)	21(4)	12(3)	16(4)	121(31)
	Total	53(16)	50(13)	64(16)	52(13)	54(16)	69(16)	45(12)	65(20)	452(122)
Forest non-Rubiaceae	10	8(2)	11(3)	9(2)	12(2)	12(2)	12(3)	8(0)	13(2)	85(16)
	25	11(1)	8(2)	10(2)	12(3)	15(4)	11(3)	8(2)	16(2)	91(19)
	50	13(1)	10(3)	5(1)	12(3)	12(3)	12(3)	8(2)	15(2)	87(18)
	100	12(0)	7(2)	15(2)	12(3)	12(3)	11(2)	8(2)	15(2)	92(16)
	Total	44(4)	36(10)	39(7)	48(11)	51(12)	46(11)	32(6)	59(8)	355(69)

Two-letter abbreviations denote codes for each site. Numbers outside parentheses denote the number of leaves analyzed visually for pathogen damage and numbers inside parentheses denote the number of pooled molecular samples for fungal community characterization. Fungal communities were characterized via fungalITS2 metabarcoding using the fungal-specific primers ITS4^57^ and 5.8SR^58^.

### Leaf imaging for damage morphotyping & seedling damage survey

The same day the leaves were collected, they were transported to the Las Cruces Biological Station in sterilized coin envelopes at ambient air temperature, sealed in plastic bags partially filled with desiccant. At the field station, we photographed each leaf against a uniform background. We manually screened each image for leaf spot incidence. Other disease symptoms, including powdery mildew, downy mildew, necrosis, and chlorosis, were also documented but were very rare and excluded from analysis in this study. After leaves were photographed, sections of each leaf were haphazardly selected, and 800 mg (fresh weight) was removed with a flame-sterilized scalpel, avoiding the midvein. Samples were dried in open 2-mL microcentrifuge tubes placed above silica gel in air-tight containers for ~12 h and transported to the Aldrich-Wolfe lab. Samples were lyophilized for 48 h at −80 °C using a FreeZone 6 Plus freeze dryer (Labconco Corporation, Kansas City, MO, USA) and subsequently stored at ambient temperature.

In late May 2022, we established ten 1 × 1 m plots over the existing seedling community in a subset of four of the eight forest sites (Supplementary Materials Table S1), starting at 5 m from the forest edge and proceeding every 10 m to 105 m into the forest. We individually tagged all woody plant species in the plot, identified them to species, and scored them for leaf fungal damage (%). We assessed the amount of leaf fungal damage as a visual proportion of total leaf damage to undamaged leaf for the entire seedling. Foliar damage was remeasured biweekly, and seedling mortality status was assessed weekly for 8 weeks. If more than 20 individuals of any species were present in a given plot, all were counted, but only a random 20 were tagged. Using these data, we calculated the total amount of foliar damage at the plot level, averaging across seedlings. We also calculated the mean phylogenetic distance from coffee at the plot level using the R package V.PhyloMaker2^55^ (version 0.1.0).

### Sampling airborne fungi

To investigate airborne propagule pressure, Modified Wilson and Cook Samplers^56^ (hereafter, air samplers) were constructed at Las Cruces Biological Station. These wind-driven devices consist of a free-moving mast-mounted pole and an aluminum sail that rotates the device with the wind. Each 1.36 m high air sampler was equipped with three wide-mouth 32-oz (946 mL) plastic jars (ULINE, #S-18072, Pleasant Prairie, Wisconsin, USA) positioned at 30, 80, and 130 cm above the soil surface to ensure sampling of air at heights in the typical range of understory shrub species. We fitted each jar with modified lids containing intake and outtake tubes for airflow oriented in relation to the sail such that the intake tube faced into the wind to capture airborne particles (Supplementary Materials; Fig. S1). Prior to field use, collection jars and lids were sterilized in 10% bleach (*v/v*) for 30 min and dried for ~45 min at 60 °C to evaporate any remaining bleach solution. After drying, the modified lids were sealed onto the jars with plastic wrap covering the intake and outtake tubes until placed in the field.

To sample airborne fungi, we placed one air sampler each at 10, 25, 50, and 100 m from the coffee-forest border in the forest understory and at 50 m from the coffee-forest border in the coffee field (Fig. 1). To compare which propagules were moving from coffee to forest and vice versa, we also placed two air samplers at the coffee-forest border on fixed poles that could not rotate, one facing the coffee and the other facing the forest (Fig. S1). Each month, deployed jars were replaced with bleach-sterilized jars in the field, and the deployed jars were sealed, transported to Las Cruces Biological Station, refrigerated, and processed within 24 h.

We rinsed the contents of the three collection jars from each air sampler with 50 mL of ultrapure water onto a 0.45 μm GN-6 nitrocellulose membrane (Metricel, St. Louis, MO, USA) on a Büchner funnel attached to a Welch WOB-L Fluid Aspiration Vacuum Pump (Chemtech Scientific, Tampa, FL, USA) to collect particulates, including fungal propagules, which are generally larger than 1 μm ^1^. This resulted in seven samples for each site each month, one for each air sampler, containing the airborne fungi accumulated over the course of the preceding month. Samples were collected at six sites in April 2021, at seven sites in May 2021, and at all eight sites in June and July 2021. In total, 203 airborne samples were collected across four months and eight sampling locations along the coffee-forest gradient. Of these, 136 yielded sufficient fungal DNA after quality control of the sequencing data. We included negative controls each time we sampled by processing an unused collection jar, sterilized and dried identically to those deployed in the field each collection day, to confirm that no substantive contamination persisted after sterilization or was introduced during filtration. We placed each airborne sample on its membrane in an individual, sterile 5-mL tube from the DNeasy® PowerWater Kit (Qiagen, Germantown MD, USA). We dried these samples by puncturing tube lids with a flame-sterilized needle and placing them inside a sealed plastic bag filled with silica gel for 24 h at 4 °C. Tubes were then sealed with new lids and stored at −20 °C. Tubes were shipped frozen on dry ice to North Dakota State University and stored at −20 °C prior to DNA isolation.

### Molecular characterization of leaf and airborne fungi

For both leaf and air samples, we added a sterile 6.35 mm chrome-steel bead (BioSpec Products, Inc., Bartlesville, Oklahoma, USA) to each sample tube. The desiccated leaf material was pulverized dry while the air samples were pulverized wet in lysis buffer (PW1), per kit instructions, using a TissueLyser II (Qiagen, Germantown, Maryland, USA) at 27 Hz for 2 m for each plate orientation (4 min total) per operating instructions.

DNA from the airborne samples was isolated using the DNeasy® PowerWater kit following the manufacturer’s protocol (Qiagen, Germantown MD, USA). We processed one blank for every 47 samples during extraction. DNA from our leaf material was isolated using the Qiagen DNeasy® Plant Kit following the manufacturer’s protocol, with the following modifications. Because we had twice the maximum amount of dried sample in individual microcentrifuge tubes ( ~ 200 mg) for the 96-well format, we doubled the amounts of AP1 and RNase and added them directly to each sample in its microcentrifuge tube in the heating step to ensure samples were at the correct concentration for adequate cell lysis and to improve RNA degradation. After vortexing, we transferred 400 μL of each lysate to its position in the 96-well plates, thereby adjusting the samples to their optimal concentration and volume and allowing us to proceed with the manufacturer’s protocol. We included the optional third ethanol wash step to further remove impurities from DNA. The DNA extracts were eluted from the column twice with 50 µL of Tris-EDTA and stored at −80 °C.

For leaves, we pooled 20 μL of DNA extract from each individual of a given species at each distance within each site into a single composite sample for that species at that distance at that site. Unfortunately, during this process, six samples were inadvertently destroyed, one coffee sample and five non-Rubiaceae samples (Table 1). Our DNA extraction controls for leaves were pooled as one sample for leaves and airborne samples, respectively. For airborne sample controls taken during field sampling (see above), those with detectable DNA by Qubit 2.0 Fluorometer with the Qubit dsDNA HS Assay Kit (Invitrogen, Thermo Fisher Scientific, Waltham, MA, USA) following the manufacturer’s protocol were included in sequencing, and control-associated OTUs were subtracted from true samples in the resulting OTU abundance table.

We shipped all eluates on dry ice to the University of Minnesota Genomic Center (UMGC, Minneapolis, MN, USA) for library preparation following our previously published protocol^57^ and sequencing of the fungal ITS2 region of the small ribosomal subunit using the fungal-specific primers ITS4^58^ (TCCTCCGCTTATTGATATGC) and 5.8SR^59^ (TCGATGAAGAACGCAGCG) with Nextera adapters (Table S9) and Illumina MiSeq™ protocol developed by UMGC^60^. Briefly, these steps involved library preparation via amplifying the target region once for each sample in a 25 µL reaction containing 12.5 µL KAPA HiFi HotStart ReadyMix (Roche, Basel, Switzerland, Catalog #: 07958927001), 0.8 µL each of forward ITS4_Nextera and reverse 5.8SR_Nextera primers (Integrated DNA Technologies, Coralville, IA, USA), 1 µL of template DNA, and nuclease-free water to volume. Thermocycling conditions were: initial denaturation at 95 °C for 3 min; 30 cycles of 98 °C for 20 s, 65.7 °C for 15 s, and 72 °C for 45 s; and a final extension at 72 °C for 5 min. Following a 1:100 dilution, samples underwent an additional ten cycles under the same conditions to incorporate Illumina flow cell adapters and unique dual indices. This two-step amplification approach minimizes amplification bias and amplicon mislabeling while improving downstream chimera detection^61^. Libraries were sequenced on an Illumina MiSeq platform using a v3 600-cycle kit to generate paired-end 2 × 300 bp reads. Negative controls were included to monitor contamination. We did not apply a separate *post hoc* correction for tag jumping/switching; however, the use of unique dual indexing and negative controls was intended to minimize the effect of such artifacts.

We processed the raw FASTA sequences for the ITS2 region via the PIPITS 3.0 bioinformatics pipeline^62^ with a 97% similarity threshold, using the UNITE v8.3 database^63^. This fully automated pipeline prepares FASTA sequences, detects and removes chimeras and sequences <100 bp in length, assigns operational taxonomic units (OTUs), designates a taxonomic prediction and a confidence score for that prediction based on known sequences from the UNITE fungal database, and generates an OTU abundance matrix. Following paired-end assembly, reads were quality-filtered using FASTQ_QUALITY_FILTER (FASTX-Toolkit) as implemented in PIPITS v3.0 (default parameters: −q 20, −p 75), retaining only reads with Phred scores ≥ 20 for ≥ 75% of bases. We chose not to rarefy the OTU table to avoid discarding data^64^. Only OTUs with an assignment of Kingdom Fungi were included in the final dataset. OTUs with confidence scores < 0.85 (with a possible range of 0–1) were considered unidentified. The highest read count for each OTU detected in the negative controls was subtracted from its read count, if any, in each of the biological samples.

### Statistical analyses

#### Visual assessment of foliar disease symptoms and fungal damage of seedlings

We conducted all analyses in R (v4.2.3)^65^ We used a two-step modeling approach to (1) determine whether leaf spot incidence was higher in coffee than in forest leaves, and (2) examine if leaf spot incidence changed with distance into the forest for forest Rubiaceae (native coffee relatives) versus forest non-Rubiaceae hosts. To test whether leaf spot incidence was greater in coffee than in forest leaves, we fitted a binomial generalized linear mixed model (GLMM) with logit link to the binary response of leaf spot presence/absence, vegetation type (coffee vs. forest) as a fixed effect, and both site and plant species as random effects to account for site-to-site variation and variable sample sizes of given species. We used the Laplace approximation to ensure stable estimation despite sparse or uneven counts^66^.

To determine whether close relatives of coffee (plants within Rubiaceae) exhibited a different pattern of edge-related leaf spot symptoms compared to more distantly related forest hosts, we classified each forest sample as belonging to either a Rubiaceae (close relatives of coffee) or a non-Rubiaceae host. We ran binomial GLMMs predicting leaf spot presence/absence as a function of the fixed effects physical distance from the coffee forest border and phylogenetic distance (described below) of the host species from coffee, plus their interaction, with site as a random effect for Rubiaceae and non-Rubiaceae forest plants. These analyses were done using the lme4 (v. 1.1-37), emmeans (v.1.11.2-8), and multcomp (v.1.4-28) R packages.

With respect to our seedling fungal damage surveys, we used a mixed linear regression model to explain the change in fungal damage at the plot level as a function of physical distance from the coffee field, phylogenetic distance from the coffee, and their interaction term with the forest site as a random effect to account for site variation. Predictions for low and high phylogenetic distance in Fig. 4 were generated using the minimum and maximum observed plot-level phylogenetic distance values in the dataset after transformation and standardization. Specifically, phylogenetic distance was first log-transformed as log(1 + phylogenetic distance), then *z*-transformed, and the fitted relationships were plotted at the minimum and maximum values of this transformed variable. This was done using the lme4 (v. 1.1-37) and lmerTest(v.3.1-3) R packages.

### Airborne fungi

Because airborne samples had extremely low biomass and were collected repeatedly at the same locations across months, amplification success varied stochastically among sampling events. We interpreted failed amplifications as reflecting very low or absent propagule loads rather than reliable zero values that should contribute equally to an average. Accordingly, read counts were summed across sampling months within each site to approximate aggregate propagule loads. To assess spatial patterns of all fungal propagules, we used a negative binomial mixed model on total fungal read counts along transects, with distance as a continuous fixed effect and site as a random effect. We then selected Pleosporales as a focal group for follow-up analysis because this order contains many known coffee-associated pathogens and constituted a large and taxonomically diverse component of the coffee leaf mycobiome in our dataset (Supplementary Materials Fig. S2 and Table S4), making it a suitable candidate group for investigating potential coffee-to-forest fungal spillover. We used a beta-binomial mixed model with a logit link to model the relative abundance of Pleosporales. Throughout this study, relative abundance was calculated as the proportion of all fungal reads in each sample belonging to the focal group. These analyses were conducted using the lme4 (v. 1.1-31) and glmmTMB (v.1.1.12) R packages^67^. We selected a beta-binomial approach because it can handle overdispersion and is commonly used to model compositional microbiome data^68^.

### Foliar fungi

To assess if host relatedness to coffee plays a role in the abundance of coffee-associated fungal taxa in the host phyllosphere, we calculated the phylogenetic distance of each of our forest plant species from coffee by acquiring sequences spanning the 18S, ITS1, 5.8S, ITS2 and 26S regions of DNA for each of the plant species used in this study or the closest relatives with relevant sequence data in GenBank^69^ (Table S3). All sequences we used contained at least a complete 5.8S segment. Phylogenetic distance from coffee for each understory plant species was extracted using the Tamura-Nei model with complete deletion via MEGA version 11.0.13^70^. The phylogenetic distance of coffee to itself was set at zero.

As with the air sampler data, we used the glmmTMB R package (v.1.1.12) to evaluate if the relative abundance of Pleosporales declined from coffee into the forest, as with the air samplers. We fitted a beta-binomial mixed model, with the relative abundance of Pleosporales as the response variable, physical distance from the coffee-forest border as a fixed effect, and site as a random effect to partition variance due to sample location. To test whether the relative abundance of Pleosporales on forest leaves was influenced by proximity to the coffee field and the host plant’s relatedness to coffee, we took a stratified approach and fitted the above model for forest Rubiaceae leaves and forest non-Rubiaceae leaves separately. Phylogenetic distance of host plants from coffee was not a strong predictor of Pleosporales relative abundance in our Rubiaceae or non-Rubiaceae forest models and was removed from our final models. For final model selection, we compared all possible combinations of our variables of interest and selected the model with the lowest Akaike Information Criterion (AIC) value^71^.

### Investigating spillover of individual taxa

To determine if individual fungal taxa from the air and leaves attenuated with distance into the forest, we first excluded taxa from the OTU matrix that appeared fewer than five times, as these would not yield a detectable signal across distances. We used generalized joint attribute modeling^72^ (GJAM) using the gjam R package (v.2.6.2) to test for fungal taxa that declined with distance into the forest interior using data from the forest air samplers (forest-facing at the border, and at 10, 25, 50, 100 m into the forest). In contrast to our GLMM approach above, in which airborne samples were summed across sampling months to approximate propagule load and exposure of a dominant clade, the multivariate GJAM analyses were conducted on unpooled data, with sampling month retained as a fixed effect. This allowed us to account for temporal variation in airborne fungal composition and resolve taxon-specific responses. We used physical distance from the coffee-forest border as a continuous fixed effect, sampling month as a categorical fixed effect, and site as a random effect. This modeling approach can accommodate multivariate datasets composed of various data types that are dominated by zeros^73^.

For forest leaf samples, the OTU matrix was too large to run in its entirety, even with the built-in dimension-reduction step in this modeling approach. Consequently, we assessed the forest patterns of the 1000 most abundant OTUs observed in coffee that also appeared on > 10 pooled leaf samples in the forest ( ~ 56% of the total reads in the leaf samples), including all other OTUs as a separate binned category in the model. We ran this full model with physical distance into the forest from the coffee-forest border and phylogenetic distance from coffee of the host plant as continuous fixed effects and site as a random effect. For both GJAM models, we used three chains of 5000 iterations and 500 burn-in iterations to achieve chain convergence using the package default flat uniform priors. Effect estimates are taxon-specific GJAM regression coefficients on the model latent scale; they describe the direction and relative strength of association between each fungal taxon and a given predictor while accounting for random effects and residual covariance among taxa. Coefficients whose posterior intervals did not overlap with zero were considered significant, indicating statistical support for a non-zero association with that predictor. Because these coefficients are expressed on the latent model scale, they are not directly interpretable as fold-changes or proportional changes in relative abundance. We matched the genera of OTUs to their ecological guild in order to determine the probable ecological role of these taxa and their potential as plant pathogens using the Primary Lifestyle designation of the FungalTraits database^74^. For OTUs that could not be assigned to a genus, we manually assigned a guild designation when possible.

### Leaf fungal alpha & beta diversity

Lastly, alpha diversity was calculated for foliar communities using included coverage-standardized richness (Hill *q* = 0) and coverage-standardized (Hill *q* = 1) diversity (Shannon-order Hill diversity) using coverage-based interpolation/extrapolation from the iNEXT r package (v.2.0.20). Group differences were tested with Kruskal-Wallis tests and BH-adjusted pairwise Wilcoxon ranked sum tests. Beta diversity was quantified and visualized in the R package vegan (v.2.7-1) from sample-by-OTU count matrices using Bray-Curtis dissimilarity. Community structure was ordinated with non-metric multidimensional scaling using a three-dimensional solution (*k* = 3) and visualized as pairwise projections of axis combinations. Group differences for host type (coffee, forest Rubiaceae, forest non-Rubiaceae) were tested with PERMANOVA (9999 permutations) for the full dataset (bray ~ host type) and for forest samples (bray ~ distance*host type), with term-wise tests (by = terms). Because distance was modeled as a continuous predictor in the forest PERMANOVA, this tested for a linear community-compositional gradient with distance. Pairwise PERMANOVAs among the three host types were performed on the full dataset and adjusted using a Benjamini-Hochberg correction. Homogeneity of multivariate dispersion was assessed with betadisper and permutest functions (9999 permutations). In forest analyses, dispersion was tested by host type and separately by a categorical distance variable (10, 25, 50, 100 m).

### Reporting summary

Further information on research design is available in the Nature Portfolio Reporting Summary linked to this article.

## Supplementary information

Supplementary Information

Reporting Summary

Transparent Peer Review file

## Data Availability

The processed sequencing, visual leaf damage, seedling survey data, and associated metdata from which the findings of this study are based are deposited on Figshare 10.6084/m9.figshare.30535862. Raw sequences generated from this study have been deposited in the NCBI Sequence Read Archive under BioProject accession PRJNA1225740.

## Code availability

Scripts to reproduce the analyses in this manuscript are deposited on Figshare: 10.6084/m9.figshare.30535862.

## References

[1] Golan, J. J. & Pringle, A. Long-distance dispersal of fungi. *Microbiol. Spectr*. **5**, 2016 (2017). 10.1128/microbiolspec.funk-0047-2016 PMC11687522 28710849

[2] Apigo, A. & Oono, R. Plant abundance, but not plant evolutionary history, shapes patterns of host specificity in foliar fungal endophytes. *Ecosphere* **13**, e03879 (2022).

[3] Pajares-Murgó, M. et al. Mutualistic and antagonistic phyllosphere fungi contribute to plant recruitment in natural communities. *J. Ecol*. **113**, 1422–1433 (2024).

[4] Pendrill, F. et al. Disentangling the numbers behind agriculture-driven tropical deforestation. *Science* **377**, eabm9267 (2022). 36074840 10.1126/science.abm9267

[5] Ma, J., Li, J., Wu, W. & Liu, J. Global forest fragmentation change from 2000 to 2020. *Nat. Commun.* **14**, 3752 (2023). 37433782 10.1038/s41467-023-39221-x PMC10336092

[6] Blitzer, E. J. et al. Spillover of functionally important organisms between managed and natural habitats. *Agric. Ecosyst. Environ.* **146**, 34–43 (2012).

[7] Spear, J. E., Grijalva, E. K., Michaels, J. S. & Parker, S. S. Ecological spillover dynamics of organisms from urban to natural landscapes. *J. Urban Ecol.* **4**, juy008 (2018).

[8] Rice, R. A. A place unbecoming: the coffee farm of Northern Latin America. *Geogr. Rev.* **89**, 554–579 (1999). 20662186

[9] Cadenasso, M. L., Pickett, S. T. A., Weathers, K. C. & Jones, C. G. A framework for a theory of ecological boundaries. *BioScience* **53**, 750–758 (2003).

[10] Benítez-Malvido, J., Lázaro, A. & Ferraz, I. D. K. Effect of distance to edge and edge interaction on seedling regeneration and biotic damage in tropical rainforest fragments: a long-term experiment. *J. Ecol.* **106**, 2204–2217 (2018).

[11] Johnson, B. L. & Haddad, N. M. Edge effects, not connectivity, determine the incidence and development of a foliar fungal plant disease. *Ecology* **92**, 1551–1558 (2011). 21905421 10.1890/10-1072.1

[12] Laurance, W. F. et al. Habitat fragmentation, variable edge effects, and the landscape-divergence hypothesis. *PLoS ONE* **2**, e1017 (2007). 17925865 10.1371/journal.pone.0001017 PMC1995757

[13] Reis Medeiros, H. et al. Landscape composition regulates the spillover of beneficial insects between forest remnants and adjacent coffee plantations. *Perspect. Ecol. Conserv.* **20**, 111–116 (2022).

[14] Gilbert, G. S. & Hubbell, S. P. Plant diseases and the conservation of tropical forests. *BioScience* **46**, 98–106 (1996).

[15] Janzen, D. H. Herbivores and the number of tree species in tropical forests. *Am. Nat.* **104**, 501–528 (1970).

[16] Connell, J. H. On the role of natural enemies in preventing competitive exclusion in some marine animals and in rain forest trees. *Dyn. Popul.* **298**, 298–312 (1971).

[17] Hopkins, S. R., Fleming-Davies, A. E., Belden, L. K. & Wojdak, J. M. Systematic review of modelling assumptions and empirical evidence: does parasite transmission increase nonlinearly with host density? *Methods Ecol. Evol.* **11**, 476–486 (2020).

[18] Wang, Y.-P. et al. Optimizing plant disease management in agricultural ecosystems through rational in-crop diversification. *Front. Plant Sci*. **12**, 767209 (2021). 10.3389/fpls.2021.767209 PMC8739928 35003160

[19] Gilbert, G. S. & Webb, C. O. Phylogenetic signal in plant pathogen–host range. *Proc. Natl. Acad. Sci. USA* **104**, 4979–4983 (2007). 17360396 10.1073/pnas.0607968104 PMC1829250

[20] Arnold, A. E. & Lutzoni, F. Diversity and host range of foliar fungal endophytes: are tropical leaves biodiversity hotspots? *Ecology* **88**, 541–549 (2007). 17503580 10.1890/05-1459

[21] Qian, X. et al. Host genotype strongly influences phyllosphere fungal communities associated with *Mussaenda pubescens* var. *alba* (Rubiaceae). *Fungal Ecol.* **36**, 141–151 (2018).

[22] Yang, T., Xiong, C., Zhou, J., Zhang, W. & Qian, X. Phyllosphere mycobiome: diversity and function. in *Plant Mycobiome: Diversity, Interactions and Uses* (eds Rashad, Y. M., Baka, Z. A. M. & Moussa, T. A. A.) 63–120 (Springer International Publishing, 2023). 10.1007/978-3-031-28307-9_4.

[23] Guo, C., Yang, A. & Zhang, W.-H. Host identity determines the bacterial and fungal community and network structures in the phyllosphere of plant species in a temperate steppe. *Phytobiomes J.* **8**, 143–154 (2024).

[24] Parker, I. M. et al. Phylogenetic structure and host abundance drive disease pressure in communities. *Nature* **520**, 542–544 (2015). 25903634 10.1038/nature14372

[25] Kembel, S. W. & Mueller, R. C. Plant traits and taxonomy drive host associations in tropical phyllosphere fungal communities. *Botany* **92**, 303–311 (2014).

[26] Arnold, A. E. et al. Fungal endophytes limit pathogen damage in a tropical tree. *Proc. Natl. Acad. Sci. USA* **100**, 15649–15654 (2003). 14671327 10.1073/pnas.2533483100 PMC307622

[27] Harvey, C. A. et al. Transformation of coffee-growing landscapes across Latin America. A review. *Agron. Sustain. Dev.* **41**, 62 (2021). 34484434 10.1007/s13593-021-00712-0 PMC8406019

[28] Caudill, S. A., Brokaw, J. N., Doublet, D. & Rice, R. A. Forest and trees: shade management, forest proximity and pollinator communities in southern Costa Rica coffee agriculture. *Renew. Agric. Food Syst.* **32**, 417–427 (2017).

[29] Delprete, P. G. & Jardim, J. G. Systematics, taxonomy and floristics of Brazilian Rubiaceae: an overview about the current status and future challenges. *Rodriguésia.* **63**, 101–128 (2012).

[30] Razafimandimbison, S. G. & Rydin, C. Phylogeny and classification of the coffee family (Rubiaceae, Gentianales): overview and outlook. *TAXON* **73**, 673–717 (2024).

[31] Garibaldi, L. A. et al. Stability of pollination services decreases with isolation from natural areas despite honey bee visits. *Ecol. Lett.* **14**, 1062–1072 (2011). 21806746 10.1111/j.1461-0248.2011.01669.x

[32] Wisler, G. C. & Norris, R. F. Interactions between weeds and cultivated plants as related to management of plant pathogens. *Weed Sci.* **53**, 914–917 (2005).

[33] Harper, K. A. et al. Edge influence on forest structure and composition in fragmented landscapes. *Conserv. Biol.* **19**, 768–782 (2005).

[34] Willmer, J. N. G., Püttker, T. & Prevedello, J. A. Global impacts of edge effects on species richness. *Biol. Conserv.* **272**, 109654 (2022).

[35] Chamberlain, S. A., Whitney, K. D. & Rudgers, J. A. Proximity to agriculture alters abundance and community composition of wild sunflower mutualists and antagonists. *Ecosphere* **4**, 1–16 (2013).

[36] Evans, D. M., Turley, N. E., Levey, D. J. & Tewksbury, J. J. Habitat patch shape, not corridors, determines herbivory and fruit production of an annual plant. *Ecology* **93**, 1016–1025 (2012). 22764488 10.1890/11-0642.1

[37] Crous, P. W., Groenewald, J. Z., Bensch, K., Gené, J. & Guarro, J. Genera of phytopathogenic fungi known from culture: 1–379. *Stud. Mycol.* **112**, 261–633 (2025). 41522876 10.3114/sim.2025.112.05 PMC12786776

[38] Oliveira, M., Delgado, L., Ribeiro, H. & Abreu, I. Fungal spores from Pleosporales in the atmosphere of urban and rural locations in Portugal. *J. Environ. Monit. JEM* **12**, 1187–1194 (2010). 21491687 10.1039/b913705j

[39] Abrego, N. et al. Airborne DNA reveals predictable spatial and seasonal dynamics of fungi. *Nature* **631**, 835–842 (2024). 38987593 10.1038/s41586-024-07658-9 PMC11269176

[40] Chen, J. et al. Fungal endophyte communities of crucifer crops are seasonally dynamic and structured by plant identity, plant tissue and environmental factors. *Front. Microbiol*. **11**, 1519 (2020). 10.3389/fmicb.2020.01519 PMC7373767 32760366

[41] Barwell, L. J. et al. Evolutionary trait-based approaches for predicting future global impacts of plant pathogens in the genus *Phytophthora*. *J. Appl. Ecol.* **58**, 718–730 (2021). 33883780 10.1111/1365-2664.13820 PMC8048555

[42] Aveskamp, M. M., De Gruyter, J., Woudenberg, J. H. C., Verkley, G. J. M. & Crous, P. W. Highlights of the Didymellaceae: a polyphasic approach to characterise *Phoma* and related pleosporalean genera. *Stud. Mycol.* **65**, 1–60 (2010). 20502538 10.3114/sim.2010.65.01 PMC2836210

[43] Deb, D., Khan, A. & Dey, N. *Phoma* diseases: epidemiology and control. *Plant Pathol.* **69**, 1203–1217 (2020).

[44] Aparecido, L. E. D. O. et al. Climate change and *Phoma* spp. Leaf spot of Arabica coffee: a CMIP6 modeling approach. *Rev. Bras. Meteorol.* **38**, e38230041 (2024).

[45] Fitt, B. D. L., McCartney, H. A. & West, J. S. Dispersal of foliar plant pathogens: mechanisms, gradients and spatial patterns. in *The Epidemiology of Plant Diseases* (eds Cooke, B. M., JONES, D. G. & KAYE, B.) 159–192 10.1007/1-4020-4581-6_6 (Springer Netherlands, 2006).

[46] Oono, R., Rasmussen, A. & Lefèvre, E. Distance decay relationships in foliar fungal endophytes are driven by rare taxa. *Environ. Microbiol.* **19**, 2794–2805 (2017). 28640496 10.1111/1462-2920.13799

[47] Rand, T. A., Tylianakis, J. M. & Tscharntke, T. Spillover edge effects: the dispersal of agriculturally subsidized insect natural enemies into adjacent natural habitats. *Ecol. Lett.* **9**, 603–614 (2006). 16643305 10.1111/j.1461-0248.2006.00911.x

[48] Power, A. G. & Mitchell, C. E. Pathogen spillover in disease epidemics. *Am. Nat.* **164**, S79–S89 (2004). 15540144 10.1086/424610

[49] Avelino, J. et al. The coffee rust crises in Colombia and Central America (2008–2013): impacts, plausible causes and proposed solutions. *Food Secur* **7**, 303–321 (2015).

[50] Kelly, D. W., Paterson, R. A., Townsend, C. R., Poulin, R. & Tompkins, D. M. Parasite spillback: a neglected concept in invasion ecology? *Ecology* **90**, 2047–2056 (2009). 19739367 10.1890/08-1085.1

[51] Schütz, L. et al. How to promote multifunctionality of vegetated strips in arable farming: a qualitative approach for Germany. *Ecosphere* **13**, e4229 (2022).

[52] Holdridge, L. R. et al. Forest environments in tropical life zones: a pilot study. *For. Environ. Trop. Life Zones Pilot Study* https://www.cabdirect.org/cabdirect/abstract/19716605728 (1971).

[53] Zahawi, R. A., Duran, G. & Kormann, U. Sixty-seven years of land-use change in southern Costa Rica. *PLoS ONE* **10**, e0143554 (2015). 26599325 10.1371/journal.pone.0143554 PMC4657907

[54] Tosi, J. A. Jr. *República de Costa Rica*: *mapa ecológico según la clasificación de zonas de vida del mundo por L. R. Holdridge*[map] (Centro Científico Tropical, San José, Costa Rica, 1969).

[55] Jin, Y. & Qian, H. V. PhyloMaker2: an updated and enlarged R package that can generate very large phylogenies for vascular plants. *Plant Divers* **44**, 335–339 (2022). 35967255 10.1016/j.pld.2022.05.005 PMC9363651

[56] Goossens, D. & Offer, Z. Y. Wind tunnel and field calibration of six aeolian dust samplers. *Atmos. Environ.* **34**, 1043–1057 (2000).

[57] White, T. J., Bruns, T., Lee, S. & Taylor, J. Amplification and direct sequencing of fungal ribosomal RNA genes for phylogenetics. In *PCR Protocols: A Guide to Methods and Applications* (eds Innis, M. A., Gelfand, D. H., Sninsky, J. J. & White, T. J.) 315–322 (Academic Press, 1990).

[58] Vilgalys, R. & Hester, M. Rapid genetic identification and mapping of enzymatically amplified ribosomal DNA from several *Cryptococcus* species. *J. Bacteriol.* **172**, 4238–4246 (1990). 2376561 10.1128/jb.172.8.4238-4246.1990 PMC213247

[59] Gohl, D. M. et al. Systematic improvement of amplicon marker gene methods for increased accuracy in microbiome studies. *Nat. Biotechnol.* **34**, 942–949 (2016). 27454739 10.1038/nbt.3601

[60] Bohmann, K. et al. Strategies for sample labeling and library preparation in DNA metabarcoding studies. *Mol. Ecol. Resour.* **22**, 1231–1246 (2022). 34551203 10.1111/1755-0998.13512 PMC9293284

[61] Sternhagen, E. C. et al. Contrasting patterns of functional diversity in coffee root fungal communities associated with organic and conventionally managed fields. *Appl. Environ. Microbiol.* **86**, e00052–20 (2020). 32220838 10.1128/AEM.00052-20 PMC7237791

[62] Gweon, H. S. et al. PIPITS: an automated pipeline for analyses of fungal internal transcribed spacer sequences from the Illumina sequencing platform. *Methods Ecol. Evol.* **6**, 973–980 (2015). 27570615 10.1111/2041-210X.12399 PMC4981123

[63] Abarenkov, K. et al. The UNITE database for molecular identification and taxonomic communication of fungi and other eukaryotes: sequences, taxa and classifications reconsidered. *Nucleic Acids Res.* **52**, D791–D797 (2024). 37953409 10.1093/nar/gkad1039 PMC10767974

[64] McMurdie, P. J. & Holmes, S. Waste not, want not: why rarefying microbiome data is inadmissible. *PLoS Comput. Biol.* **10**, e1003531 (2014). 24699258 10.1371/journal.pcbi.1003531 PMC3974642

[65] R Core Team. R: a language and environment for statistical computing. (R Core Team, 2024).

[66] Rue, H., Martino, S. & Chopin, N. Approximate Bayesian inference for latent Gaussian models by using integrated nested Laplace approximations. *J. R. Stat. Soc. Ser. B Stat. Methodol.* **71**, 319–392 (2009).

[67] Bates, D. et al. Lme4: linear mixed-effects models using ‘Eigen’ and S4. (CRAN, 2025).

[68] Martin, B. D., Witten, D. & Willis, A. D. Modeling microbial abundances and dysbiosis with beta-binomial regression. *Ann. Appl. Stat.* **14**, 94–115 (2020). 32983313 10.1214/19-aoas1283 PMC7514055

[69] Benson, D. A. et al. GenBank. *Nucleic Acids Res.* **46**, D41–D47 (2018). 29140468 10.1093/nar/gkx1094 PMC5753231

[70] Kumar, S., Stecher, G., Li, M., Knyaz, C. & Tamura, K. MEGA X: molecular evolutionary genetics analysis across computing platforms. *Mol. Biol. Evol.* **35**, 1547–1549 (2018). 29722887 10.1093/molbev/msy096 PMC5967553

[71] Burnham, K. P. & Anderson, D. R. Multimodel inference: understanding AIC and BIC in model selection. *Sociol. Methods Res.* **33**, 261–304 (2004).

[72] Clark, J. S., Nemergut, D., Seyednasrollah, B., Turner, P. J. & Zhang, S. Generalized joint attribute modeling for biodiversity analysis: median-zero, multivariate, multifarious data. *Ecol. Monogr.* **87**, 34–56 (2017).

[73] Taylor-Rodríguez, D., Kaufeld, K., Schliep, E. M., Clark, J. S. & Gelfand, A. E. Joint species distribution modeling: dimension reduction using Dirichlet processes. *Bayesian Anal.* **12**, 939–967 (2017).

[74] Põlme, S. et al. FungalTraits: a user-friendly traits database of fungi and fungus-like stramenopiles. *Fungal Divers.* **105**, 1–16 (2020).

