# Supplementary material for: Field-to-forest fungal spillover is a biotic edge effect of coffee in Costa Rica
Source: Nat Commun. 2026 Jul 24;17:9025. doi: 10.1038/s41467-026-75178-3 (PMC13507213; doi:10.1038/s41467-026-75178-3)
Supplement: Supplementary file 1 [file 41467_2026_75178_MOESM2_ESM.pdf]

nature portfolio

Corresponding author(s): Jeffrey A Lackmann

Last updated by author(s): May 26, 2025

Reporting Summary

Nature Portfolio wishes to improve the reproducibility of the work that we publish. This form provides structure for consistency and transparency in reporting. For further information on Nature Portfolio policies, see our Editorial Policies and the Editorial Policy Checklist.

Statistics

For all statistical analyses, confirm that the following items are present in the figure legend, table legend, main text, or Methods section.

n/a	Confirmed	
[ ]	[x]	The exact sample size (*n*) for each experimental group/condition, given as a discrete number and unit of measurement
[ ]	[x]	A statement on whether measurements were taken from distinct samples or whether the same sample was measured repeatedly
[ ]	[x]	The statistical test(s) used AND whether they are one- or two-sided<br>*Only common tests should be described solely by name; describe more complex techniques in the Methods section.*
[ ]	[x]	A description of all covariates tested
[ ]	[x]	A description of any assumptions or corrections, such as tests of normality and adjustment for multiple comparisons
[ ]	[x]	A full description of the statistical parameters including central tendency (e.g. means) or other basic estimates (e.g. regression coefficient) AND variation (e.g. standard deviation) or associated estimates of uncertainty (e.g. confidence intervals)
[ ]	[x]	For null hypothesis testing, the test statistic (e.g. *F*, *t*, *r*) with confidence intervals, effect sizes, degrees of freedom and *P* value noted<br>*Give P values as exact values whenever suitable.*
[ ]	[x]	For Bayesian analysis, information on the choice of priors and Markov chain Monte Carlo settings
[x]	[ ]	For hierarchical and complex designs, identification of the appropriate level for tests and full reporting of outcomes
[x]	[ ]	Estimates of effect sizes (e.g. Cohen's *d*, Pearson's *r*), indicating how they were calculated

Our web collection on statistics for biologists contains articles on many of the points above.

Software and code

Policy information about availability of computer code

Data collection	No code was used to collect data from other sources. All data was generated by the authors.
Data analysis	All code and an explanatory README file have been deposited on Figshare. Analysis was conducted in RStudio (v.4.2.3) with use of the following essential packages:<br>lme4 (v. 1.1-37)<br>glmmTMB (v.1.1.12)<br>iNEXT (v.2.0.20)<br>gjam (v.2.6.2)<br>vegan (v.2.7-1)<br>lmerTest(v.3.1-3)<br><br>All other packages for data wrangling, etc. nclude<br>ggplot2 (v.4.0.0)<br>ggeffects (v.2.3.1)<br>readxl (v.1.4.5)<br>tidyverse (v.2.0.0)<br>dplyr (v.1.2.0)<br>car (v.3.1-3)<br>multcomp (v.1.4-28)<br>scales (v.1.4.0)

broom.mixed (v.0.2.9.6)
purr (v.1.1.0)
V.PhyloMaker2 (v.0.1.0)
emmeans (v.1.11.2-8)

For manuscripts utilizing custom algorithms or software that are central to the research but not yet described in published literature, software must be made available to editors and reviewers. We strongly encourage code deposition in a community repository (e.g. GitHub). See the Nature Portfolio guidelines for submitting code & software for further information.

Data

Policy information about availability of data

All manuscripts must include a data availability statement. This statement should provide the following information, where applicable:

- Accession codes, unique identifiers, or web links for publicly available datasets
- A description of any restrictions on data availability
- For clinical datasets or third party data, please ensure that the statement adheres to our policy

The processed sequencing, visual leaf damage, seedling survey data, and associated metdata from which the findings of this study are based are deposited on Figshare https://doi.org/10.6084/m9.figshare.30535862. Raw sequences generated from this study have been deposited in the NCBI Sequence Read Archive under BioProject accession PRJNA1225740 [https://www.ncbi.nlm.nih.gov/bioproject/PRJNA1225740].

Research involving human participants, their data, or biological material

Policy information about studies with human participants or human data. See also policy information about sex, gender (identity/presentation), and sexual orientation and race, ethnicity and racism.

Reporting on sex and gender	*Use the terms sex (biological attribute) and gender (shaped by social and cultural circumstances) carefully in order to avoid confusing both terms. Indicate if findings apply to only one sex or gender; describe whether sex and gender were considered in study design; whether sex and/or gender was determined based on self-reporting or assigned and methods used. Provide in the source data disaggregated sex and gender data, where this information has been collected, and if consent has been obtained for sharing of individual-level data; provide overall numbers in this Reporting Summary. Please state if this information has not been collected. Report sex- and gender-based analyses where performed, justify reasons for lack of sex- and gender-based analysis.*
Reporting on race, ethnicity, or other socially relevant groupings	*Please specify the socially constructed or socially relevant categorization variable(s) used in your manuscript and explain why they were used. Please note that such variables should not be used as proxies for other socially constructed/relevant variables (for example, race or ethnicity should not be used as a proxy for socioeconomic status). Provide clear definitions of the relevant terms used, how they were provided (by the participants/respondents, the researchers, or third parties), and the method(s) used to classify people into the different categories (e.g. self-report, census or administrative data, social media data, etc.) Please provide details about how you controlled for confounding variables in your analyses.*
Population characteristics	*Describe the covariate-relevant population characteristics of the human research participants (e.g. age, genotypic information, past and current diagnosis and treatment categories). If you filled out the behavioural & social sciences study design questions and have nothing to add here, write "See above."*
Recruitment	*Describe how participants were recruited. Outline any potential self-selection bias or other biases that may be present and how these are likely to impact results.*
Ethics oversight	*Identify the organization(s) that approved the study protocol.*

Note that full information on the approval of the study protocol must also be provided in the manuscript.

Field-specific reporting

Please select the one below that is the best fit for your research. If you are not sure, read the appropriate sections before making your selection.

☐ Life sciences ☐ Behavioural & social sciences ☒ Ecological, evolutionary & environmental sciences

For a reference copy of the document with all sections, see nature.com/documents/nr-reporting-summary-flat.pdf

Ecological, evolutionary & environmental sciences study design

All studies must disclose on these points even when the disclosure is negative.

Study description	We sampled leaves and fungal aerobiota along transects at eight privately owned coffee farms in southern Costa Rica adjacent to forest fragments. In this adjacent forest we sampled understory plants within and outside the family Rubiaceae and assessed visual symptoms of disease alongside characterizing the foliar mycobiome via metabarcoding using the ITS2 region. This was done in an effort to investigate if high-density monocultures directly adjacent for fragmented forests influence the foliar mycobiome of understorty trees or changes pathogen dynamics near the border especially those closely related to the crop plant.

Research sample	There were two primary research samples for this study. The first was a leaf from the second fully expanded leaf pair counting from a randomly selected branch tip f coffee and forest Rubiaceae and non Rubiaceae understory plants. The second were the monthly aggregate total airborne fungal propagules at a given sampling distance that corresponded to the distance from which a leaf was sampled. At each sampling distance in the forest(10, 25, 50 and 100 m from the coffee-forest border) we attempted to sample leaves from three Rubiaceae and two non-Rubiaceae plant species (avoiding monocots).
Sampling strategy	We attempted, whenever possible to sample 1 leaf from four individual plants of each species at each distance, sampling within a ~5 m radius of the prescribed sampling distance along the transect (marked with flagging). This sampling size was calculated because it allowed us to sample 5 different understory species at each distance, have >3 replicates at each distance for mean and variance, and abide within the budgetary limits of the study with respect to downstream processing costs (amplicon library preparation, etc...) Our sampling scheme was such that we wanted if at all possible, comparable sampling effort between coffee and each understory plant species. We felt this was appropriate given our focus on screening forest understory foliar mycobiomes from various species relative to coffee fields. While this meant that the sampling effort at the broader level coffee and forest at large were imbalanced, we felt it more appropriate to maintain comparability at the level of individual species rather than comparing coffee to forest leaves at large.
Data collection	JAL collected all leaves using a flame sterilized scissors cutting at the juncture of the base and the petiole. Leaves were placed in sterile coin envelopes which were collected into open plastic ziploc bags. A sample number was ascribed to each leaf in a field notebook with the prefix JL (JL001, JL002, etc...) which was later transcribed to an Excel spreadsheet. These leaves were identified to species in the field and this plant ID was confirmed by FOB at Las Cruces Biological Station using representative material collected by JAL and LAW. JAL transported this material to Las Cruces Biological Station for immediate processing that afternoon. JAL photographed the leaves with a ruler in the frame of the image to calibrate leaf area. Leaves were placed on uniform blue background for uniform contrast during image analysis. EPR and JF collected the aerobiota bottles as described in the Methods section of the manuscript. EPR processed these bottles and filtered the fungal propagules via membranes and a vacuum pump at Las Cruces Biological Station the same day they were collected from the field. All data was recorded in lab notebooks and was later transcribed to digital spreadsheets with two additional people (lab technicians) confirming the handwritten values were transcribed to the digital spreadsheets without error. BB and GE recorded seedling damage bi-weekly for eight weeks using field notebooks and transcribing field data to digital spreadsheets. Leaf DNA was extracted by JAL and EPR extracted aerbiota membrane DNA. Extraction details can be found in the methods section of the manuscript. All subsequent amplicon data was managed by JAL and LAW.
Timing and spatial scale	All fieldwork from preparation to execution was conducted March 2nd to August 10th 2021. The aerobiota membranes were taken from each site one month apart from each sampling date Starting in late April/early may - Early August 2021. These sampling dates were on the Monday and Wednesday of each week. Exact sampling dates can be found in the metadata associated with the membrane matrices on the Figshare repository (membrane_order_wmeta.xlsx). As an example, aerobiota membranes would be sampled from sites 1 and 2 on Monday and Wednesday of the first week of the month. Aerobiota sampling for sites 3 & 4 would take place Monday and Wednesday of the following week each month and so on. Leaves were collected from each site one time over the course of one day. EP: June 1, LZ: June 9, DV: Jun 14, AJ: Jun 16, VP: June 21, RN June 23, AN: June 28, RB Jul 5. Co-authors BB and GE conducted the seedling survey in 2022 at a subset of our 4 sites (RN, RB, AN, LZ) from May - July. Exact dates of surveys and repeat surveys can be found in the DATEi column in the data_all_growth_phylo.csv file uploaded on the Figshare repository associated with this manuscript. In terms of spatial scale, all transects were such that they extended 100 m into the forest (100 m from any forest edge) and 100 m into the coffee (100 m from any coffee edge). There was one exception to this. The coffee plantation at RN was not wide enough to accomodate 100 m without getting <100 m from the opposite edge. In this case, the transect was 50 m shorter in the coffee (it only extended 50 m instead of 100) and we doubled our sampling effort at this distance to maintain the roughly the same number of sampled leaves in the coffee as the other sites.
Data exclusions	All leaf tissue from Virola sebifera virtually completely failed to amplify during amplicon sequencing. Each of these samples had fewer than 150 reads, roughly the same number of reads as our extraction blanks. We excluded this plant species from molecular analysis. Virola sebifera's capacity to inhibit DNA isolation is intriguing and warrants attention.
Reproducibility	Field and lab methods have been reported in sufficient detail to reproduce the methodology employed in the study and GPS coordinates from each sampling distance at each site were taken so that, should someone want to reproduce the study, the could in theory return to the exact locations at each site where leaves were sampled and spore samplers were deployed. Any modifications made to DNA extraction protocols were clearly delineated in the methods. However, to our knowledge. no replication effort has been made at these sites since the study was conducted.
Randomization	Leafs were taken randomly from each plant by using a random number generator to determine A) which branch to select, and B) which leaf to select from the leaf pair. In the coffee the random number generator was also used to select which coffee tree to select from by assigning candidate plants at each distance a number and taking from the plant that corresponded with the number from the random number generator. Samples were randomly selected for sets of DNA extractions using a random number generator.
Blinding	Blinding was not relevant for this study.

Did the study involve field work? ☒ Yes ☐ No

Field work, collection and transport

Field conditions	All sites are in the canton of Coto Brus in southern Costa Rica: Rainfall in the region where all the sites are located receives mean annual rainfall of 4000 mm and a mean annual temperature of 21 °C (Holdridge & Grenke, 1971; Zahawi et al., 2015).
Location	Site Lat-Long coordinates and elevation are listed here. By request of the farmers to keep the exact location of their land private, we have not provided precise lat-long coordinates. These, however, can be made available to reviewers should they request them.<br>Site Lat Long Elevation<br>AN: 8.906; -82.901; 839.95

LZ: 8.973 -82.927: 1193.46
DV: 8.959;-82.919; 1181.59
AJ: 8.924; -82.935 ;1031.31
RN: 8.886; -82.851; 1181.4
VP: 8.940;-82.890; 1173.85
RB: 8.845;-82.901; 990.46
EP: 8.973; -82.809; 1125.46

Access & import/export	Our sampling in Costa Rica and subsequent DNA analysis was approved by the Comisión Nacional para la Gestión de la Biodiversidad (resolution No. R-004-2021-OT-CONAGEBIO).
Disturbance	Care was taken not to disturb the sites beyond sampling leaves and establishing the spore collectors. When traversing the transect we avoided moving or cutting any plant or treefall material. All field equipment was sterilized by soaking in bleach prior to fieldwork at each site at Las Cruces Biological Station or, in the case of sampling scissors via ethanol flame sterilization between each sampled leaf. Any equipment including flagging, bags, notebooks, wire, etc... was removed from the field after sampling. Any time anything needed to be processed prepared in the field, we did so in a pre-existing cultivation related prep area footpath near to the the coffee field to avoid spending excess time in the forest or coffee plantation .

Reporting for specific materials, systems and methods

We require information from authors about some types of materials, experimental systems and methods used in many studies. Here, indicate whether each material, system or method listed is relevant to your study. If you are not sure if a list item applies to your research, read the appropriate section before selecting a response.

Materials & experimental systems

n/a	Involved in the study	
☒	☐	Antibodies
☒	☐	Eukaryotic cell lines
☒	☐	Palaeontology and archaeology
☒	☐	Animals and other organisms
☒	☐	Clinical data
☒	☐	Dual use research of concern
☐	☒	Plants

Methods

n/a	Involved in the study	
☒	☐	ChIP-seq
☒	☐	Flow cytometry
☒	☐	MRI-based neuroimaging

Dual use research of concern

Policy information about dual use research of concern

Hazards

Could the accidental, deliberate or reckless misuse of agents or technologies generated in the work, or the application of information presented in the manuscript, pose a threat to:

No	Yes	
☒	☐	Public health
☒	☐	National security
☒	☐	Crops and/or livestock
☒	☐	Ecosystems
☒	☐	Any other significant area

Experiments of concern

Does the work involve any of these experiments of concern:

No	Yes	
☒	☐	Demonstrate how to render a vaccine ineffective
☒	☐	Confer resistance to therapeutically useful antibiotics or antiviral agents
☒	☐	Enhance the virulence of a pathogen or render a nonpathogen virulent
☒	☐	Increase transmissibility of a pathogen
☒	☐	Alter the host range of a pathogen
☒	☐	Enable evasion of diagnostic/detection modalities
☒	☐	Enable the weaponization of a biological agent or toxin
☒	☐	Any other potentially harmful combination of experiments and agents

Plants

Seed stocks

No seed stocks was used. All plant sampling was of plants naturally ocurring at the field sites.

Novel plant genotypes

Describe the methods by which all novel plant genotypes were produced. This includes those generated by transgenic approaches, gene editing, chemical/radiation-based mutagenesis and hybridization. For transgenic lines, describe the transformation method, the number of independent lines analyzed and the generation upon which experiments were performed. For gene-edited lines, describe the editor used, the endogenous sequence targeted for editing, the targeting guide RNA sequence (if applicable) and how the editor was applied.

Authentication

Describe any authentication procedures for each seed stock used or novel genotype generated. Describe any experiments used to assess the effect of a mutation and, where applicable, how potential secondary effects (e.g. second site T-DNA insertions, mosiacism, off-target gene editing) were examined.
