# Supplementary material for: Field-to-forest fungal spillover is a biotic edge effect of coffee in Costa Rica
Source: Nat Commun. 2026 Jul 24;17:9025. doi: 10.1038/s41467-026-75178-3 (PMC13507213; doi:10.1038/s41467-026-75178-3)
Supplement: Supplementary file 2 [file 41467_2026_75178_MOESM3_ESM.pdf]

nature portfolio

Peer Review File

Field-to-forest fungal spillover is a biotic edge effect of coffee in Costa Rica

Corresponding Author: Dr Jeffrey Lackmann

This manuscript has been previously reviewed at another journal. This document only contains information relating to versions considered at Nature Communications.

This file contains all reviewer reports in order by version, followed by all author rebuttals in order by version.

Version 0:

Reviewer comments:

Reviewer #1

(Remarks to the Author)
Thanks to Lackmann et al for their nice manuscript on phyllosphere fungal communities on coffee and neighboring forest understory species. I enjoyed reviewing the manuscript and think it will make a good contribution to the literature.
I think it will be acceptable for publication after minor/moderate revisions.
I have made specific comments in the annotated manuscript attached. I lay out my main criticisms below.
good luck with the resubmission.
Introduction
Im not sure if the Janzen Connell is really a useful theory to hang your manuscript onto. I found in places that the hypothesis only confused me about how it was relevant and if it even mattered. there is plenty of literature out there on the effects of edges and on fungal host range specificity to make sense of your work, without trying to fit your results to a theory developed for vertebrates/invertebrates.
Methods
you do not give much detail on the sites locations, or on the composition of the sites. I cannot picture what the sites look like.
you also need to detail if the coffee sites received fungicide treatments which could affect your results.
you should define relative abundance.
results
several comments in the manuscript.
Discussion
my major criticism is that you havent given enough evidence to indicate the movement direction of the fungal spores. how do you know that the fungal pathogens aren't spilling over form the forest into the coffee? your main hypothesis is that coffee acts as a reservoir for fungi to move into forest plants. why couldnt it be the other way around?
your discussion often repeats the results, with little interpretation. some paragraphs (437-449; 462-472; 496-504; 514-520) have no references. you should be making sense of your results with reference to other published works in your discussion.
finally, you should carefully review your bibliography as i found a few references were missing.

Reviewer #2

(Remarks to the Author)
The authors Lackmann et al. have reported that coffee fields act as a reservoir for foliar fungi, which influences the fungal communities in neighbouring forests. Leaves across varying distances at 8 coffee-forest borders in Costa Rica were screened for disease symptoms, showing the incidence of leaf damage declining with distance into the forest. Over 900 leaf and air samples were collected along the coffee-forest transects for sequencing of the ITS2 region. The most abundant coffee-associated OTUs declined with distance from the border.

Overall, the manuscript is very clear and well presented, the fungal community profiling is performed up the standards of the field, the design and data analysis are elegant, and the conclusions very interesting and supported by the data. A few things require further explanation or discussion, and I would suggest adding alpha and beta diversity analysis which are standard

for the field and will help with understanding the microbial ecology at play. I would thus recommend major revision, but the additional analysis should be easy enough to do.

I assume the authors have deposited their sequences to a public database and made all their script and metadata publicly available, but no information was present in the manuscript, so I was not able to verify that.

I detail below suggestions to improve the manuscript:

Methods section:
- L110 How far were the sites from each other? Providing a map of the sites would help.
- L122: I would normally say that a single leaf per individual is not enough to capture the composition of the leaf microbiota of an individual. The fact that the authors do find a signal in their data with one leaf per individual do raises the question of how the leaf was selected, where in the plant (e.g. middle canopy, top of the canopy, north facing, south facing etc.), and how did the authors ensured that their leaf choice was not biased (for instance by selecting a more spotted leaf when closer to the coffee field).
- L141: Following up on my previous comment, I would assume the leaf spot incidence to be variable between leaves of a given individual. How did the author ensure an unbiased selection of the single leaf per individual?
- L157: I really like the seedling survey, it is really great!
- L178-179: the two air samplers facing two directions are also really cool and clever!
- L225: give the details of the PCR protocol so that readers have all the information without having to look into another paper.
- L225: Also precise which read length was sequenced (e.g. PE250 for paired-end 250 bp).
- L225: detail the primary quality control. Were reads filtered based on their fastq quality scores?
- L226: explain how the multiplexing was conducted. Did you use tagged primers? If so, detail that. Also, has tag jumping/switching been assessed and corrected?
- Figure 1: The leaf discs are misleading in this figure. We usually take leaf discs to normalise samples based on the leaf surface, but you normalised samples based on weight (which is also fine!). I would suggest finding another representation, unless you actually did sample discs in which case you need to explain it in the methods.
- L308: I don’t understand why you did not add the host species as a random factor here.
- L370: More clarification is required to understand how the model distinguished between species which were closely and distantly related to coffee.

Results
I would start the result section (or a supplementary material/extended data section) with a classical analysis of alpha (e.g. Shannon index) and beta (e.g. Bray-Curtis distance visualised as an ordination plot) diversity in coffee versus forest leaves, maybe focusing on Rubiaceae. Even if it is general, it would be informative to know how different the fungal diversity is between the two, and maybe how it changes with distance.
Figure 3: Add a panel to this figure with a simple box plot to allow easy comparison of leaf spot incidence between coffee and forest.
L345 and for all further models: Give the full model results in (supplementary) tables, including estimates and p-values for all parameters.
Figure 4: Describe in the method section how you got the prediction for high and low phylogenetic distance, i.e. which phylogenetic distance values did you use as high and low for prediction of fungal damage/distance relationship.
Figure 6: I am not an expert in GJAM, could you explain the biological signification of the estimated effect size? Can we say which order of magnitude in relative abundance changes does this represent?

Discussion:
L466: More context and information on Pleosporales should be given, and their relevance in answering the questions addressed in this study should be discussed.
L477 – 479: Could you explain a little bit more this argument? I am not sure if I am following.
L499: You cannot claim that from read counts, especially on rarefied data. You need to do a proper alpha diversity analysis.
L504 – 512: I really like this paragraph and the idea suggested by the authors

Minor comments:
- L68: Precise what you mean by “steep biotic gradient”
- L161: give reference and version for the package
- L201: wet with what?
- L280: Explain what you did to pool samples. Did you do a mean of OTU read counts? Or a sum?
- L284: Do you mean that you summed all reads belonging to Pleosporales and calculated their relative abundance?
- Figure 2B did you mean richness of OTUs? Maybe rephrase by “number of OTUs” so it is clear what the numbers mean.
- L344: Figure 3 instead of 3A
- L397: ‘Respectively’ spelling error
- L409: I only see 6 OTU classified as pathogens on Figure 8A, not 10. Which categories do you consider as pathogens?
- L471: full stop missing

Reviewer #3

(Remarks to the Author)

I co-reviewed this manuscript with one of the reviewers who provided the listed reports. This is part of the Nature Communications initiative to facilitate training in peer review and to provide appropriate recognition for Early Career Researchers who co-review manuscripts.

Version 1:

Reviewer comments:

Reviewer #1

(Remarks to the Author)
Thanks for considering my previous comments and making changes to the manuscript.
I recommend your manuscript for publication. I look forward to seeing it published.
Richard O'Hanlon

Reviewer #2

(Remarks to the Author)
The authors have made considerable efforts to revise the manuscript, and we thank them for responding to our recommendations.
• Introduction is clear, outlines the aims and hypothesis of the study. Gives clear, relevant, and well referenced background information.
• Methods section provides much more detail, including information on the study site, leaf selection, and the PCR program. We completely understand why the authors cannot provide a map, and this does not prevent the reader from understanding the results.
• Results now include alpha and beta diversity analysis which we think help drawing their conclusion
• Discussion is easy to follow and logically explains conclusions.

We have a few minor comments:
• Figure 3 – It's much clearer how phylogenetic distance has been calculated, but a colour scale key should be added the plot.
• This clarification from figure 6, "negative values denote distance into coffee from coffee-forest edge" should also be added to figure 3?
• L493 Figure 7 is miss labelled as figure 8.

We also have a few concerns about reproducibility, as for now, the scripts have not been made public yet. Please provide a link to your repository for the reviewing stage (it can be a private link if needed).
In addition, the data available in the FigShare repository cannot be reused in its current state as it lacks major information. I would suggest adding a README file which contains:
- the title summary of the article
- a description of each file, including each column of the file (just a general description for the OTU columns is fine of course).

Reviewer #3

(Remarks to the Author)
I co-reviewed this manuscript with one of the reviewers who provided the listed reports. This is part of the Nature Communications initiative to facilitate training in peer review and to provide appropriate recognition for Early Career Researchers who co-review manuscripts.

Open Access This Peer Review File is licensed under a Creative Commons Attribution 4.0 International License, which permits use, sharing, adaptation, distribution and reproduction in any medium or format, as long as you give appropriate credit to the original author(s) and the source, provide a link to the Creative Commons license, and indicate if changes were made.
In cases where reviewers are anonymous, credit should be given to 'Anonymous Referee' and the source.
The images or other third party material in this Peer Review File are included in the article's Creative Commons license, unless indicated otherwise in a credit line to the material. If material is not included in the article's Creative Commons license and your intended use is not permitted by statutory regulation or exceeds the permitted use, you will need to obtain permission directly from the copyright holder.
To view a copy of this license, visit https://creativecommons.org/licenses/by/4.0/

General Comments from Authors:
We thank the reviewers for taking the time to review this manuscript. We think the revisions have greatly strengthened this study, especially in the Discussion, which has been substantially reworked to improve organization and better lay out our central arguments. During the revision process numbers changed substantially from the original reviewer comments. We have tried to provide the line numbers from the revised manuscript (with track changes visible) associated with the comments to direct the reviewers on how we've addressed their concerns. These line numbers are very different from the original manuscript and the new manuscript without track changes. We felt this was easiest for reviewers to see where we've made changes and how we've responded to their concerns.

Throughout this document Reviewer comments will be in black, our responses in blue.

REVIEWER 1:

Thanks to Lackmann et al for their nice manuscript on phyllosphere fungal communities on coffee and neighboring forest understory species. I enjoyed reviewing the manuscript and think it will make a good contribution to the literature.

Thank you. We will address both the general comments here and the line-by-line comments from the attached file immediately after.

I think it will be acceptable for publication after minor/moderate revisions.
I have made specific comments in the annotated manuscript attached. I lay out my main criticisms below.
good luck with the resubmission.

Thank you for taking the time to review this manuscript. We appreciate your thoughtful feedback.

Introduction
Im not sure if the Janzen Connell is really a useful theory to hang your manuscript onto. I found in places that the hypothesis only confused me about how it was relevant and if it even mattered. there is plenty of literature out there on the effects of edges and on fungal host range specificity to make sense of your work, without trying to fit your results to a theory developed for vertebrates/invertebrates.

We agree that Janzen-Connell (JC) theory was not originally formulated in the context of plant-associated fungi, nor at crop–forest interfaces. Janzen and Connell independently developed the framework to explain density-dependent mortality driven by natural enemies in tropical trees and marine invertebrates, respectively. Our intention was not to suggest that JC directly predicts the patterns observed here, but rather to use it as an analogous framework for thinking about density-dependent fungal accumulation associated with a dominant host, which in this case is coffee. We found it conceptually useful for framing hypotheses about how high host density at

the agricultural edge might structure pathogen pressure and spillover dynamics in a way that is mechanistically separate from the logic of a diversity-based spillover pool from field to forest .

That said, we see the reviewer's point that invoking JC may distract from the more directly relevant literature on edge effects and fungal host-range specificity we discuss here. In response, we have revised the Introduction (lines 89-90) to clarify that JC is presented as an analogy rather than a strict theoretical anchor. We now foreground edge ecology, and host specificity as the primary conceptual foundations of the study, especially in the discussion.

Methods

you do not give much detail on the sites locations, or on the composition of the sites. I cannot picture what the sites look like. You also need to detail if the coffee sites received fungicide treatments which could affect your results.

We've added table **S1** with latitude and longitude coordinates for each site, left imprecise enough to protect the anonymity of the landowners, which was a condition for being able to conduct this study on their land. As such, we've refrained from adding a map of site locations to protect their privacy. We've added details to the site descriptions as well as supplemental Figure **S3** and Table **S2** to give a sense of the understory plant composition in these sites. We've also clarified in the methods that all coffee was conventionally managed and that fungicides have been employed during management.

you should define relative abundance.

Thank you for pointing out that relative abundance was not formally defined in the original submission. We now explicitly define relative abundance as the proportion of sequencing reads assigned to a given focal group divided by the total number of reads per sample following quality filtering and processing (i.e., total-sum scaling of the feature table). This definition is now included in the Methods section (lines 455-456).

results

several comments in the manuscript.

Discussion

my major criticism is that you havent given enough evidence to indicate the movement direction of the fungal spores. how do you know that the fungal pathogens aren't spilling over form the forest into the coffee? your main hypothesis is that coffee acts as a reservoir for fungi to move into forest plants. why couldnt it be the other way around?

We see the reviewer's point that the previous draft did not frame this study well enough to explicitly illustrate that we are not implying that the forest-to-field spillover is not happening. We hope that the way the paper is now framed in the introduction and discussion more clearly emphasizes that we are not implying unidirectionality of spillover but rather calling attention to bidirectionality that has largely been unexplored in the existing spillover literature.

With respect to the direction of fungal movement or origin, the reviewer's comment is an important one. We cannot, from this experiment, definitively point to a source of origin for the fungi we detected. We have made that clear in the discussion (Lines 1108-1250). However, that is not what we set out to test, nor is it, in our mind, the essential question here. It is not always whether a particular fungus originates in the coffee or forest that is important with respect to the impact of spillover from one habitat to the other. In this case, we suspected that the highly dense monoculture of coffee amplifies certain fungi that may then impact adjacent forest plants in ways they otherwise wouldn't encounter if they were not proximate to the monoculture. For example, a fungus may naturally occur in wild forests. But when this fungus encounters the coffee, due to the density of the plantation, if it can make use of this plant as a habitat or resource, it would then sporulate at much higher densities (proportionate to the density of its suitable host) than it ever would in the much more diverse forest understory. We've added this idea to discussion (lines 1093-1107). This creates propagule pressure from the coffee into the adjacent forest, creating a dynamic, in this case, where that fungus is augmented near to the forest border. We suspect this kind of spillback is likely here. Fungi may also be coffee-specific that then move to the forest. With respect to what we tested, the origin does not matter with respect to the effect at the forest border. We have substantially revised and reframed the discussion to make this more explicit.

your discussion often repeats the results, with little interpretation. some paragraphs have no references. you should be making sense of your results with reference to other published works in your discussion.

We agree. Because of this, we have substantially revised the discussion to avoid this, to integrate more literature, to avoid unnecessary repetition, and to more appropriately contextualize our results with the broader literature. We believe that the manuscript is much stronger as a result and thank the reviewer for this comment.

finally, you should carefully review your bibliography as i found a few references were missing. (FROM text) Reviewer comment: Not in references (Janzen, 1970; Connell, 1971)

We apologize for these omissions. We have ensured that these references are now in the bibliography. We have, to the best of our knowledge, ensured that every reference is listed in the bibliography.

We have moved "plants" for clarity per the reviewer's suggestion (now line 98)

Reviewer comment (now line 126): Your hypothesis testing seems to take for granted that fungi would move from coffee to forest, and not that forests would act as a reservoir of fungi that could infect coffee.

Thank you for this comment. It was not our intention to imply that forests do not act as reservoirs for fungi that infect coffee. We agree that forest-to-field spillover occurs. We

attempted to acknowledge this dynamic in lines 70–73, but we appreciate that the framing may not have made this sufficiently clear.

To address this concern, we have revised the Introduction (see in particular lines 83-87) to more explicitly state that our study was intentionally framed to examine spillover from field to forest, not because the reverse does not occur, but because forest-to-crop spillover has been studied more. In contrast, the potential for high-density crop systems to generate or amplify pathogen reservoirs that spill over into adjacent natural ecosystems has received much less attention.

Our central contention is that intensive monoculture systems house crop-augmented reservoirs of organisms that can influence nearby forest communities. This dynamic represents an underexplored dimension of spillover ecology, one in which dense low-diversity reservoirs have their own capacity to influence nearby environments. We believe we have clarified this framing in the revised manuscript so that it is not perceived as taking for granted or ignoring spillover from forest to field.

Reviewer comment: Were the heights/age of the sampled plants roughly the same?

We have added the height criteria for plant sampling (Line 185). Understory plants > 50 cm in height and with accessible leaves (below ~2 m).

Reviewer comment: Will you list the species of the plants in the supplementary. It would be useful for others who might want to reuse the sequencing data to know which hosts you sampled.

Plant species are included in the supplement in Table S7

Reviewer comment (Now line 200): Can you provide some images of the types of disease you assessed on coffee and non-coffee plants? In supplementary docs

Since leaf spot is the only disease we quantified for that particular analysis, we've added indicative pictures of this disease morphotype on various species of leaves that we sampled (Fig. S2).

Reviewer comment (Now line 182): This isn't figure 1

This carryover typo from a previous draft has been removed.

Reviewer comment (now line 213): Can you identify which sites these were?

We have added a supplementary table with site information that now includes a column that specifies which sites we conducted the seedling survey at. See table **S1**.

Reviewer comment (now line 250): How do you know that the spores didn't enter via the exhaust tube in the trap?

Thank you for this question. Indeed, we never clarified this. A sentence has been added to figure S1 (the diagram of the samplers to explain this . Briefly here, airflow through the trap is governed by pressure differentials and resistance along the flow path. The intake and exhaust tubes were of unequal length, with the exhaust tube being longer. Because airflow resistance increases with tube length, this design creates a preferential direction of exchange: air entering the trap follows the lower-resistance pathway, while air exits via the higher-resistance tube.

For spores to enter through the exhaust tube, a sustained reversal of the pressure gradient would be required. Under ambient field conditions, passive airflow is driven by natural convection and wind-induced pressure differences, which favor directional exchange rather than bidirectional mixing through both tubes simultaneously.

Occasional stochastic noise via momentary turbulence cannot be entirely excluded, but the physical design of the trap makes systematic entry of spores through the exhaust tube very unlikely.

Reviewer comments (now lines 450-453). This is results so should come in the results section not in methods
And Fig. 2 - Move to results. Out of place in the methods

Thank you for the suggestion. Because the dominance of Pleosporales is used to justify the analytical focus of the subsequent model, we believe some of this information is appropriate in the Methods as part of the rationale for selecting Pleosporales as a focal taxonomic group. But we agree that presenting data in a figure in the methods is inappropriate. We no longer treat the pie chart as a main result, have moved the figure to the Supplementary Materials (Fig. S2) and revised the text accordingly.

Reviewer Comment in text: Is this data on phylogenetic distance index included in supplementary

Yes, phylogenetic distance scores are included in table S3 in the "Pdist" column. The genomic regions from which the phylogenetic distance was calculated are included in the table caption.

Reviewer suggested edits have been integrated throughout to improve the clarity of the language here

Reviewer comment: [How we calculate relative abundance] not explained in methods.

We have now defined relative abundance in the methods (Lines 455-456): "Throughout this study relative abundance was calculated as the proportion of all fungal reads in each sample belonging to the focal group."

Reviewer comment: How many OTUs of this group [forest and air Pleosporales] did you find. You focus a lot on this group so it would be good to know how OTU rich the group was.

We've added these details in text. 932 OTUs in forest leaves and 542 in the airborne dataset (Lines 807, 855)

Reviewer Comment: (Now lines X-Y): What is the logic of thinking of excluding the border forest samplers? Surely you should just include them and not mention the option to exclude them?

This comparison was included as a sensitivity analysis to determine whether the observed distance trend was driven solely by elevated Pleosporales abundance at the forest edge. By re-running the regression using only forest-interior samplers (excluding the border samplers), we tested whether the decline persisted beyond the immediate edge environment. The trend remained significant, indicating that the pattern reflects attenuation with distance into the forest rather than being leveraged exclusively by the edge samples.

We've added some clarifying language to help contextualize this robustness test. It now reads "This pattern remained significant when the analysis was restricted to forest-interior samplers, indicating that the decline was not driven solely by elevated abundance at the forest edge (Table S8)." Lines 808-810

Reviewer comment (now line 873-900 on to the end of the section): How can you tell that this relationship isn't that the further you get phylogenetically, the less physical distance matters?

Thank you for raising this point. Because interaction terms are symmetric, the pattern could indeed be described in either direction. In our models, the main effect of host phylogenetic distance was negative while the interaction between spatial distance and phylogenetic distance was positive. This coefficient structure indicates that the negative association between fungal abundance and host phylogenetic distance becomes progressively weaker with increasing distance from coffee. Equivalently, it could be interpreted that the spatial distance effect is strongest on hosts closely related to coffee and diminishes on more distantly related hosts. Importantly, both descriptions reflect the same interaction surface and are consistent with the same ecological interpretation: that the signal associated with coffee declines with increasing distance into the forest and is most pronounced on hosts most closely related to coffee. We have revised the manuscript text to clarify this interpretation starting at line 873.

NOTE: With respect to the reviewer's comments for the discussion, we have made a substantial revision here and so line to line comments are less helpful than simply pointing out how we addressed this concern in the new draft.

Reviewer Comment**:** This can be true, but it could also be true that forests can act as a reservoir for coffee fungi.

Which part of your data shows that the greatest risk is to the forest plants rather than to the coffee when these two land uses are in close proximity?

We've made clearer now that bidirectionality of spillover is the central framework into which our results fit. Hopefully this makes it clear that we are not implying that forests are not reservoirs for coffee fungi. With respect to the second part of this comment, other than the visual surveys of disease, we do not measure plant performance here. It is outside the scope of this study to empirically determine which plants are most at risk here. We simply demonstrate that adjacent coffee can impose risk via spillover to adjacent forest plants.

Line 1038: We have accepted the reviewer's suggestion, changing the naming of Pleosporales from the "dominant order of coffee leaves" to "most abundant and diverse order"

Reviewer Comment: This statement doesn't have any link to your hypothesis. In fact it could indicate that coffee is the plant under threat when a coffee plantation and forest are adjacent to each other.

This comment was regarding pointing out the high incidence of coffee disease as itself evidence of spillover of disease. We agree that this was not sufficient evidence. Higher disease incidence in coffee alone does not establish directional spillover and could reflect elevated risk within the plantation. We have revised the discussion to emphasize that the critical evidence relevant to our hypothesis with respect to visual symptoms of disease is the *combination* of higher disease incidence observed in coffee and the spatial pattern of disease incidence among forest hosts. Specifically, among forest Rubiaceae, the probability of leaf spot declines with distance from the coffee-forest border. It is this *combination* that matches the expected spatial signature of field-to-forest spillover. In other words, while higher disease burden in coffee could reflect plantation-level amplification, the distance-decay pattern observed in forest plants provides spatial evidence consistent with our hypothesis that coffee can function as a source of disease pressure for adjacent forest hosts.

Reviewer Comment: This seems like a pretty small level of support for your hypothesis. Just 10%. – This comment is in regards to the 10% of the 1,000 most abundant OTUs that show this phylogenetic signal being more abundant on closer relatives of coffee.

We agree that 10% may appear modest when framed as a simple proportion of total OTUs. However, the relevant comparison is not against 100%, but against the null expectation of random host association across phylogenetic distance. Under such a null, only a very small fraction of taxa would be expected to show consistent increases in abundance with decreasing phylogenetic distance to coffee.

That approximately 10% of the 1,000 most abundant OTUs (<50% of the total reads) exhibit this structured phylogenetic pattern represents a non-random and biologically meaningful signal.

Importantly, these OTUs are drawn from the most abundant taxa in the community, which are likely to contribute disproportionately to transmission dynamics and disease pressure. Thus, even if the majority of taxa show no such relationship, a consistent subset of abundant fungi tracking host-relatedness provides support for phylogenetically structured spillover risk.

Reviewer Comment: How can you be sure that this isn't what your data is showing? – This comment is regarding the possibility of forest to field spillover.

We agree that spillover across agro–forest interfaces is likely bidirectional, and we do not exclude the possibility that forests act as reservoirs that influence coffee in this system. We do not assert unidirectionality, but emphasize that previous work has largely focused on natural ecosystems influencing adjacent agricultural systems, whereas our analyses explicitly evaluate the reciprocal pathway. We have substantially revised the discussion to clarify this framing.

Reviewer Comment: This is largely repetition of lines 441-446 with little addition to the manuscript. This can be removed/shortened

We agree, especially with the modifications made to the previous sections. This paragraph has been removed in the restructured manuscript. Moreover, this comment brought to our attention some other repetitive elements of the discussion which prompted us to restructure the discussion. We believe it reads much better now and the manuscript is much stronger as a result. Thank you for this critique.

Reviewer comment (Now line 961): Better to say “only driver”?

We’ve rewritten this sentence with what we think is more precise language.

“If disease were driven only by microclimatic edge effects, we would expect a similar spatial response across host lineages.**”**

Reviewer comment: Why not use more directional spore traps instead of expensive and complicated isotopes?

Directional spore trapping represents a valuable and potentially more tractable approach for assessing fungal movement across habitat boundaries. We have revised the manuscript to broaden this statement and include directional spore trapping and strain-resolved genomic approaches as complementary tools for tracking fungal dispersal and confirming spillover directionality. See lines 1110-1112.

Reviewer Comment: I don’t think the survival is the important knowledge gap, it is what fungi are on these plants and comparing those with fungi on coffee

We see your point. While we contend plant survivorship is important to understand how these effects impact forest plant communities, we think that the discussion as a whole makes this point and this explicit sentence was not needed. We have amended this sentence accordingly in incorporate the reviewer’s suggestion See line 1249-1250**.**

Comment: Supplement Table S6 - There were a lot more (double?) samples taken from coffee related plants than non-coffee related plants. Has this been controlled for in the analysis and interpretation.

Thank you for raising this important point. Addressing potential confounding factors arising from our sampling design was central to determining our analytical approach. We therefore took two complementary steps to account for this issue.

First, for the leaf-spot and Pleosporales models, we used a stratified modeling framework, analyzing Rubiaceae and non-Rubiaceae forest leaves in separate models. This approach served two purposes. It controlled for sampling imbalance between close and distant coffee relatives, ensuring that any detected signal of relatedness (or interaction between relatedness and distance from the coffee–forest border) was not driven by overrepresentation of closely related hosts. In a non-stratified model, it would be difficult to disentangle biological effects from artefacts of uneven sampling.

Importantly, stratification also allowed us to test whether spatial or relatedness patterns differed between the “ingroup” (Rubiaceae, closely related to coffee) and “outgroup” (non-Rubiaceae) hosts. Using only continuous phylogenetic distance would assume a single smooth response across all hosts. By modeling the two groups separately, we were able to evaluate whether spillover dynamics or spatial gradients operated differently within and outside coffee’s primary phylogenetic neighborhood. Indeed, we found evidence that these groups exhibited distinct spatial and relatedness patterns supporting the value of this stratified approach.

Second, in the GJAM and seedling survey analyses—where all forest leaves were modeled simultaneously at the individual OTU level—we addressed the issue differently. Rather than using a categorical Rubiaceae vs. non-Rubiaceae distinction, we incorporated continuous phylogenetic distance from coffee as a predictor. This placed all samples along a continuous axis of relatedness, avoiding discrete group imbalance while retaining the full dataset.

We chose to use all forest samples together for the GJAM analysis because it models individual OTUs, where statistical power is lower and signals are inherently subtler. In contrast, the leaf-spot and Pleosporales models involved aggregated taxa, making those signals more robust to reduced sample sizes under stratification.

For the seedling survey, the sampling design differed: all seedlings within plots were sampled, meaning this dataset was not structured around ingroup/outgroup comparisons and was not subject to the same imbalance in relatedness to coffee. In this case, modeling continuous phylogenetic distance was therefore the most appropriate and biologically meaningful approach.

To assist with keeping the many models straight we’ve added a summary table of these models to assist with it. See table S8. We’ve also modified the methods to improve the clarity and justification for the modeling approaches we chose.

OTHER COMMENTS FROM REVIEWER 1:

You don’t give the location details of the sites. Can you give the lat and long
Latitude and longitude have been added to the supplementary materials Table S1.

Will you publish raw data as a supplementary file?

Yes, raw files are already deposited on figshare and will be made publicly available once the manuscript is published. Raw sequences have also been submitted to NCBI (submission ID PRJNA1225740) and will be made public upon publication. This information will go into the data availability statement if the manuscript is accepted

Line 861: Is this percentage of the whole dataset or just the 1000 most abundant OTUS

We’ve clarified this was precentage of the full dataset.

Line 503: Can you say anything about the stationary traps at the border? Did these show any fungi that were more frequent moving in one direction vs the other? It would be good to know if e.g. Pleosporales mostly moved into the forest rather than out of the forest

We’ve added the following to the discussion. “Notably, our border samplers facing coffee and forest did not show differences in total airborne propagule load, despite the greater diversity of the forest phyllosphere. We interpret this to suggest that neither side of the interface is denser with fungal propagules overall, but that coffee disproportionately contributes particular taxa to the local airborne pool as we observed with Pleosporales.” Line 1044-1048.

Comment: Lots of fungi can cause leaf spots, so this needs to be caveated.

This was with respect to leaf spot leaf spot survey results, we’ve made clear not to link this to any particular taxa. We’ve also caveated in the discussion that there are many leaf spot-causing fungi just within the order Pleosporales. See lines 1039-1042

Lines 582-591: Several comments here took issue with our use of “odds” and “incidence” rather than abundance.

These results come from the binomial model of leaf spot presence or absence with a logit link on the leaves that we sampled, not abundance of any amplicon data. We think that this issue with the language we chose raised by the reviewer was made in error. We contend that this odds and incidence are correct language statistically. However, we have made it clearer in both the methods and in this section that this is from the visual damage dataset and not the amplicon data to avoid confusion.

REVIEWER 2:

Comment: How far were the sites from each other? Providing a map of the sites would help.

We agree that information on site spacing is important. However, we assured the private landowners who granted access to their coffee farms that their identities and exact farm locations would remain confidential. To balance data transparency with this commitment, we now provide site coordinates rounded to three decimal places, which indicate approximate locations (within 100 m) while preserving anonymity. We chose not to include a site map, as even an approximate overlay on satellite imagery could compromise the confidentiality we promised participants. If the reviewers or editors ultimately consider a map essential for publication, we could provide one with deliberately coarse spatial resolution and oversized symbols to obscure exact site positions. We believe latitude and longitude coordinates serve as a reasonable compromise.

Comment:L165: I would normally say that a single leaf per individual is not enough to capture the composition of the leaf microbiota of an individual. The fact that the authors do find a signal in their data with one leaf per individual do raises the question of how the leaf was selected, where in the plant (e.g. middle canopy, top of the canopy, north facing, south facing etc.), and how did the authors ensured that their leaf choice was not biased (for instance by selecting a more spotted leaf when closer to the coffee field).

Comment: L165-185: Following up on my previous comment, I would assume that leaf spot incidence to be variable between leaves of a given individual?

We thank the reviewer for raising these two important points. We agree that foliar microbial communities and leaf spot incidence can vary among leaves within an individual plant. In designing our sampling protocol; however, our goal was not to characterize within-individual heterogeneity, but rather to obtain a standardized, representative snapshot of the foliar mycobiome at the species level while maximizing the number of individual plants' sampled across the forest–coffee gradient.

To reduce potential sampling bias, leaf selection was standardized to the second fully expanded leaf or leaf pair on each sampled individual. In addition, branch selection within each plant was randomized in the field using a predefined scheme to avoid bias in leaf selection,

We have now clarified this rationale in the manuscript and added details of the randomization procedure (lines 165-185)

Line 216: I really like the seedling survey, it is really great!

Thank you.

Line 250: The two air samplers facing two directions are also really cool and clever

Thank you.

Comment (Now line 214): give the details of the PCR protocol so that readers have all the information without having to look into another paper.

Comment: Also precise which read length was sequenced (e.g. PE250 for paired-end 250 bp).

Comment:explain how the multiplexing was conducted. Did you use tagged primers? If so, detail that. Also, has tag jumping/switching been assessed and corrected?

The above three comments are regarding the section beginning now at line 314

To address these three above comments, we've added the following details to the methods (Now Lines 270-283) "Briefly, ITS2 was amplified in 25 µL reactions containing 12.5 µL KAPA HiFi HotStart ReadyMix (Roche), 0.8 µL each of forward (ITS4_Nextera) and reverse (5.8SR_Nextera) primers, 1 µL template DNA, and nuclease-free water to volume. Thermocycling conditions were: initial denaturation at 95°C for 3 min; 30 cycles of 98°C for 20 s, 65.7°C for 15 s, and 72°C for 45 s; and a final extension at 72°C for 5 min. Following a 1:100 dilution, samples underwent an additional 10 cycles under the same conditions to incorporate Illumina flow cell adapters and unique dual indices. This two-step amplification approach minimizes amplification bias and amplicon mislabeling while improving downstream chimera detection (Bohmann *et al.,* 2022). Libraries were sequenced on an Illumina MiSeq platform using a v3 600-cycle kit to generate paired-end 2 × 300 bp reads. Negative controls were included to monitor contamination. We did not apply a separate *post hoc* correction for tag jumping/switching; however, the use of unique dual indexing and negative controls was intended to minimize such artifacts."

Comment:(now line 336): detail the primary quality control. Were reads filtered based on their fastq quality scores?

We have added the following details (Lines 290-292): "Following paired-end assembly, reads were quality filtered using FASTQ_QUALITY_FILTER (FASTX-Toolkit) as implemented in PIPITS v3.0 (default parameters: −q 20, −p 75), retaining only reads with Phred scores ≥ 20 for ≥75% of bases."

Comment: Figure 1: The leaf discs are misleading in this figure. We usually take leaf discs to normalise samples based on the leaf surface, but you normalised samples based on weight (which is also fine!). I would suggest finding another representation, unless you actually did sample discs in which case you need to explain it in the methods.

We have modified the figure to more accurately depict the process by which we extracted material and pooled DNA extracts

Comment: Now lines 443-479: I don't understand why you did not add the host species as a random factor here.

There appears to be a misunderstanding. Lines 443-479 describes the model we constructed for *airborne* fungal material captured with our MWAC samples. Thus there would be no host plants to incorporate into the model. We've added details here as to how these reads were pooled as per a later comment.

Comment: I believe this comes from the Fig. 4 caption (which is now figure 3 - the seedling survey model). More clarification is required to understand how the model distinguished between species which were closely and distantly related to coffee.

The model was fitted using average phylogenetic distance to coffee as a covariate interacting with distance from coffee plantation. The interaction was significant, and the figures show examples for two average phylogenetic distances to coffee: the closest and most distantly observed at the plot level. We have added clarifying details to the figure caption.

Results

Reviewer Comment: I would start the result section (or a supplementary material/extended data section) with a classical analysis of alpha (e.g. Shannon index) and beta (e.g. Bray-Curtis distance visualised as an ordination plot) diversity in coffee versus forest leaves, maybe focusing on Rubiaceae. Even if it is general, it would be informative to know how different the fungal diversity is between the two, and maybe how it changes with distance.

We agree that this could be useful for future work. We've taken the approach of adding this to the supplement (Supplementary Analysis and Figure S4) as we find it auxiliary to the primary findings. The paper is already fairly long so we felt it best to reference this in the supplement. We reference it at lines 857-858

Reviewer Comment: Figure 3 (now Figure 2): Add a panel to this figure with a simple box plot to allow easy comparison of leaf spot incidence between coffee and forest.

We've added this as panel A in the figure. Thank you. This simple change made the figure much better.

Reviewer Comment: for all models: Give the full model results in (supplementary) tables, including estimates and p-values for all parameters.

We have now included these details in the supplement. It is Table S8. GJAM models have their own summary tables already included (Tables S5-S7).

Reviewer Comment: Figure 4: Describe in the method section how you got the prediction for high and low phylogenetic distance, i.e. which phylogenetic distance values did you use as high and low for prediction of fungal damage/distance relationship.

We've added the following details to the methods section (Lines 416-441): "Predictions for "low" and "high" phylogenetic distance in Figure 3 were generated using the minimum and maximum observed plot-level phylogenetic distance values in the dataset after transformation and standardization. Specifically, phylogenetic distance was first log-transformed as log(1 + phylogenetic distance), then z-transformed, and the fitted relationships were plotted at the minimum and maximum values of this transformed variable." We've also added the following to figure caption: "Solid lines show model-predicted relationships from a mixed-effects model with site as a random effect, plotted at the minimum and maximum observed values of plot-level phylogenetic distance after log(1 + x) transformation and z-standardization."

Reviewer Comment:Figure 6: I am not an expert in GJAM, could you explain the biological significance of the estimated effect size? Can we say which order of magnitude in relative abundance changes does this represent?

We've added the following to the methods section (lines 566-573): "Effect estimates are taxon-specific GJAM regression coefficients on the model latent scale; they describe the direction and relative strength of association between each fungal taxon and a given predictor while accounting for random effects and residual covariance among taxa. Coefficients whose posterior intervals did not overlap zero were considered significant, indicating statistical support for a non-zero association with that predictor. Because these coefficients are expressed on the latent model scale, they are not directly interpretable as fold-changes or proportional changes in relative abundance."

We have also added clarifying language to Figures 5 & 7

Discussion:

More context and information on Pleosporales should be given, and their relevance in answering the questions addressed in this study should be discussed.

We have added more context with respect to Pleosporales ecology and dispersal to the reworked discussion (See Lines 1037-1048)

Could you explain a little bit more this argument? I am not sure if I am following. NOTE: This comment is regarding a section that has been moved regarding decoupling of signals in air and leaves.

We've revised this argument to be clearer It is the second paragraph of the discussion. Lines 953-1036

You cannot claim that from read counts, especially on rarefied data. You need to do a proper alpha diversity analysis.

NOTE: This was in response to an inference we drew from the airborne samples and read counts alone.

Thank you for raising this point. While we did not rarefy, we do agree that read counts from sequencing data cannot be interpreted as evidence of propagule load. We have therefore revised this section to instead frame the argument using diversity patterns (from the added alpha diversity analysis) in the leaf fungal communities themselves and the proper diversity analysis in the supplement, which we believe provides a more appropriate line of evidence anyway. We appreciate the reviewer drawing attention to this issue.

Reviewer Comment: Now lines 1097-1111: I really like this paragraph and the idea suggested by the authors

Thank you.

Minor comments:

Reviewer Comment: L69-70: Precise what you mean by “steep biotic gradient”

This was opaque language. We’ve rephrased it to improve clarity (Lines 69-70) to the following: “High-density monocultures like coffee plantations often generate stark biotic borders between managed and natural biotic communities”

Reviewer Comment: Now lines 225-226: give reference and version for the package

This sentence now reads, “We also calculated the mean phylogenetic distance from coffee at the plot level using the R package V.PhyloMaker2 (version 0.1.0; Jin & Qian, 2022).”

Reviewer Comment: Now lines 281: wet with what?

We’ve added the following details “The desiccated leaf material was pulverized dry while the air samples were pulverized wet in lysis buffer (PW1), per kit instructions, using a TissueLyser II (Qiagen, Germantown, Maryland, USA) at 27 Hz for two minutes for each plate orientation (4 min total) per operating instructions.

Reviewer Comment: L280: Explain what you did to pool samples. Did you do a mean of OTU read counts? Or a sum?

Reviewer Comment: L284: Do you mean that you summed all reads belonging to Pleosporales and calculated their relative abundance?

These two comments are addressed together. Yes, for this analysis we summed the sequence counts of all OTUs assigned to the order Pleosporales within each sample and modeled the

resulting proportion of Pleosporales reads relative to the total fungal reads in that sample. We have revised the text to make this clearer. See lines 443-447

Reviewer Comment: Figure 2B (now S4) did you mean richness of OTUs? Maybe rephrase by “number of OTUs” so it is clear what the numbers mean.

Corrected accordingly. Figure has been moved to the supplement. (Fig. S4).

Reviewer Comment: Now 583: Figure 3 instead of 3A.

Corrected in text. But this is now Fig. 2.

Reviewer Comment: Now Fig 5: ‘Respectively’ spelling error

Corrected in text.

Reviewer Comment: (Now line 863): I only see 6 OTU classified as pathogens on Figure 8A, not 10. Which categories do you consider as pathogens?

Thank you for catching this error. The original count inadvertently included taxa that were also represented in the interaction panel (Figure 8C), which led to double-counting across categories. We have now corrected the text accordingly. In Figure 8A, we count as pathogens only taxa assigned to the categories Plant pathogen and Plant pathogen/Endophyte, which yields 7 OTUs, not 10. We chose this revised count to avoid confusion and double-counting, and because taxa showing the interaction effect are more appropriately discussed in relation to Figure 8C, where that pattern is more ecologically informative.

Reviewer Comment: L471: full stop missing

Good catch! But the revised manuscript no longer contains this sentence.

REVIEWER 3:

I co-reviewed this manuscript with one of the reviewers who provided the listed reports. This is part of the Nature Communications initiative to facilitate training in peer review and to provide appropriate recognition for Early Career Researchers who co-review manuscripts.

Thank you for taking the time to review this manuscript.

Added References:

Bohmann K, Elbrecht V, Carøe C, Bista I, Leese F, Bunce M, Yu DW, Seymour M, Dumbrell AJ, Creer S. Strategies for sample labelling and library preparation in DNA metabarcoding studies. Mol Ecol Resour. 2022 May;22(4):1231-1246. doi: 10.1111/1755-0998.13512.

Tosi, J.A. (1969). Mapa ecoloìgico de la Repuìblica de Costa Rica. Centro Científico Tropical. https://collections.lib.uwm.edu/digital/collection/agdm/id/5376/

Jin, Y., & Qian, H. (2023). U. PhyloMaker: An R package that can generate large phylogenetic trees for plants and animals. *Plant Diversity*, *45*(3), 347-352.

Oono, R., Rasmussen, A., & Lefèvre, E. (2017). Distance decay relationships in foliar fungal endophytes are driven by rare taxa. *Environmental Microbiology*, *19*(7), 2794–2805. https://doi.org/10.1111/1462-2920.13799

Fitt, B. D. L., McCartney, H. A., & West, J. S. (2006). Dispersal of foliar plant pathogens: Mechanisms, gradients and spatial patterns. In B. M. Cooke, D. G. JONES, & B. KAYE (Eds.), *The Epidemiology of Plant Diseases* (pp. 159–192). Springer Netherlands. https://doi.org/10.1007/1-4020-4581-6_6

Barwell, L. J., Perez-Sierra, A., Henricot, B., Harris, A., Burgess, T. I., Hardy, G., Scott, P., Williams, N., Cooke, D. E. L., Green, S., Chapman, D. S., & Purse, B. V. (2021). Evolutionary trait-based approaches for predicting future global impacts of plant pathogens in the genus Phytophthora. *Journal of Applied Ecology*, *58*(4), 718–730. https://doi.org/10.1111/1365-2664.13820

Oliveira, M., Delgado, L., Ribeiro, H., & Abreu, I. (2010). Fungal spores from Pleosporales in the atmosphere of urban and rural locations in Portugal. *Journal of Environmental Monitoring: JEM*, *12*(5), 1187–1194. https://doi.org/10.1039/b913705j

Abrego, N., Furneaux, B., Hardwick, B., Somervuo, P., Palorinne, I., Aguilar-Trigueros, C. A., Andrew, N. R., Babiy, U. V., Bao, T., Bazzano, G., Bondarchuk, S. N., Bonebrake, T. C., Brennan, G. L., Bret-Harte, S., Bässler, C., Cagnolo, L., Cameron, E. K., Chapurlat, E., Creer, S., … Ovaskainen, O. (2024). Airborne DNA reveals predictable spatial and seasonal dynamics of fungi. *Nature*, *631*(8022), 835–842. https://doi.org/10.1038/s41586-024-07658-9

Chen, J., Akutse, K. S., Saqib, H. S. A., Wu, X., Yang, F., Xia, X., Wang, L., Goettel, M. S., You, M., & Gurr, G. M. (2020). Fungal Endophyte Communities of Crucifer Crops Are Seasonally Dynamic and Structured by Plant Identity, Plant Tissue and Environmental Factors. *Frontiers in Microbiology*, *11*. https://doi.org/10.3389/fmicb.2020.01519

Kelly, D. W., Paterson, R. A., Townsend, C. R., Poulin, R., & Tompkins, D. M. (2009). Parasite spillback: A neglected concept in invasion ecology? *Ecology*, *90*(8), 2047–2056. https://doi.org/10.1890/08-1085.1

Avelino, J., Cristancho, M., Georgiou, S., Imbach, P., Aguilar, L., Bornemann, G., Läderach, P., Anzueto, F., Hruska, A. J., & Morales, C. (2015). The coffee rust crises in Colombia and Central America (2008–2013): Impacts, plausible causes and proposed solutions. *Food Security*, *7*(2), 303–321. https://doi.org/10.1007/s12571-015-0446-9

Chamberlain, S. A., Whitney, K. D., & Rudgers, J. A. (2013). Proximity to agriculture alters abundance and community composition of wild sunflower mutualists and antagonists. *Ecosphere*, *4*(8), 1–16. https://doi.org/10.1890/ES13-00026.1

Evans, D. M., Turley, N. E., Levey, D. J., & Tewksbury, J. J. (2012). Habitat patch shape, not corridors, determines herbivory and fruit production of an annual plant. *Ecology*, *93*(5), 1016–1025. https://doi.org/10.1890/11-0642.1

Power, A. G., & Mitchell, C. E. (2004). Pathogen Spillover in Disease Epidemics. *The American Naturalist*, *164*(S5), S79–S89. https://doi.org/10.1086/424610

Rand, T. A., Tylianakis, J. M., & Tscharntke, T. (2006). Spillover edge effects: The dispersal of agriculturally subsidized insect natural enemies into adjacent natural habitats. *Ecology Letters*, *9*(5), 603–614. https://doi.org/10.1111/j.1461-0248.2006.00911.x

Schütz, L., Wenzel, B., Rottstock, T., Dachbrodt-Saaydeh, S., Golla, B., & Kehlenbeck, H. (2022). How to promote multifunctionality of vegetated strips in arable farming: A qualitative approach for Germany. *Ecosphere*, *13*(9), e4229. https://doi.org/10.1002/ecs2.4229

Additional changes

During revision we identified and corrected some minor inconsistencies in the Methods and Results. Neither substantively affects any central result, and both have been corrected in the manuscript text.

In the Methods we had inadvertently omitted a description of the host species random effect, although this term was included in the models used for the analysis. We have amended the Methods to reflect the full model specification. In addition, some statistical reports were carried over from exploratory analyses conducted in another statistical software. Final analyses were conducted in R, and the statistics reported in the revised manuscript now reflect the reproducible R workflow used for the study. The numerical values differ slightly between software implementations, but these differences do not change any results or inferences. Lastly, the total number of reads for foliar fungal dataset was taken from a table not completely through QC steps by mistake. We've fixed this error.

Code and Data availability:

All R scripts required to reproduce these analyses will be deposited on a Github repository under the account ja-lackmann. All data required to run these analyses will be made public on a Figshare, where they have already been deposited. Raw sequences have been submitted to NCBI These will be made public upon publication (Searchable ID: PRJNA1225740).

REVIEWERS' COMMENTS

Reviewer #1 (Remarks to the Author):

Thanks for considering my previous comments and making changes to the manuscript.
I recommend your manuscript for publication. I look forward to seeing it published.
Richard O'Hanlon

Thank you for taking the time to provide such careful and constructive feedback.

Reviewer #2 (Remarks to the Author):

The authors have made considerable efforts to revise the manuscript, and we thank them for responding to our recommendations.

• Introduction is clear, outlines the aims and hypothesis of the study. Gives clear, relevant, and well referenced background information.

Thank you.

• Methods section provides much more detail, including information on the study site, leaf selection, and the PCR program. We completely understand why the authors cannot provide a map, and this does not prevent the reader from understanding the results.

Thank you

• Results now include alpha and beta diversity analysis which we think help drawing their conclusion

Thank you. Per editorial request we have moved the alpha and beta diversity results to the main results. The associated figures remain in the supplement.

• Discussion is easy to follow and logically explains conclusions.

Thank you

We have a few minor comments:

• Figure 3 – It's much clearer how phylogenetic distance has been calculated, but a colour scale key should be added the plot.

Thank you for pointing that out. We have added a color scale key to the plot to better delineate what the colors represent

• This clarification from figure 6, "negative values denote distance into coffee from coffee-forest edge" should also be added to figure 3?

This appears to be a misunderstanding The seedling damage survey was conducted entirely in the forest. The x axis is On Fig. 3, x-axis values are *z*-standardized distances that represent observations closer to the coffee side relative to the dataset mean distance. In this

way, they do not represent the same thing that negative values denote in Fig. 6. We have added the clarifying language below to the figure caption

Axes show standardized variables: x = z-standardized distance from the coffee border and y = z-standardized log-transformed mean fungal damage of seedlings in each plot (log(1 + damage)). Negative values indicate observations below the overall mean

• L493 Figure 7 is miss labelled as figure 8.
Apologies. We've corrected this error.

We also have a few concerns about reproducibility, as for now, the scripts have not been made public yet. Please provide a link to your repository for the reviewing stage (it can be a private link if needed).
In addition, the data available in the FigShare repository cannot be reused in its current state as it lacks major information. I would suggest adding a README file which contains:
- the title summary of the article
- a description of each file, including each column of the file (just a general description for the OTU columns is fine of course).

Thank you for this reminder. We apologize for this oversight. The code and README file have been added to the Figshare repository. We've opted to keep everything centralized in one repository rather than hosting the data on Figshare and the code on GitHub.

Reviewer #3 (Remarks to the Author):

I co-reviewed this manuscript with one of the reviewers who provided the listed reports. This is part of the Nature Communications initiative to facilitate training in peer review and to provide appropriate recognition for Early Career Researchers who co-review manuscripts.

Thank you.

Final comment: In our final statistical check we found two reported statistics to be in error. The GLMM of all leaf Pleosporales was misreported when transcribing it. We have reported the correct statistical information here (see table S8 and the figure caption of Fig. 6). The results in this figure 6 caption now reads: "The relative abundance of Pleosporales decreased with distance into the forest from the coffee-forest border in our model of all molecular leaf samples (Wald $\chi^2_{(1,217)} = 27.90$, $p = 1.28 \times 10^{-7}$, $\beta_{distance} = -0.0041$, 95% CI [−0.0056, −0.0026]) and with distance from the coffee-forest border in forest Rubiaceae leaves (Wald $\chi^2_{(1,118)} = 11.45$, $p = 0.0007$, $\beta_{distance} = -0.0044$, 95% CI [−0.0069, −0.0018];" Lines 842-846 of the clean manuscript document

We also noticed the wrong model output for the border-only airborne samplers. This was an error that was directly contradicted by the discussion. Upon closer inspection we found again the statistical output had been mistranscribed. We have fixed this. While it does change the result, the result is now in agreement with the discussion, which was informed by the correct result. We apologize for any confusion.

It now reads: "At the coffee-forest border, there was no difference in read counts between air samplers facing the forest or coffee (Wald $\chi^2_{(1,10)}$ = 0.6989, p = 0.4032, $\beta_{distance}$ = 1.2358, 95% CI = [−1.6615, 4.1332])." Lines 119-122 of the clean manuscript document.
