# Supplementary material for: Field-to-forest fungal spillover is a biotic edge effect of coffee in Costa Rica
Source: Nat Commun. 2026 Jul 24;17:9025. doi: 10.1038/s41467-026-75178-3 (PMC13507213; doi:10.1038/s41467-026-75178-3)
Supplement: Supplementary file 3 [file 41467_2026_75178_MOESM1_ESM.docx]

**Supplementary Materials:** **Field-to-forest fungal spillover is a biotic edge effect of coffee in Costa Rica**

**Figure S1.** An example of Modified Wilson and Cook Samplers (MWACS)^1^ used in the study. A free-spinning mast-mounted pole (1) is welded to the wind sail (2) oriented downwind from the intake-tube (3) of three air samplers also anchored to the pole. Collection jars were positioned at 30, 80, and 130 cm above the soil surface. Intake and exhaust tubing were unequal in length inside the collection jar to create directional airflow resistance that minimized the likelihood of spore entry via the exhaust port.

**Fig S2.** Representative images of leafspot incidence on sampled leaves of four species of Rubiaceae.

**Figure S3.** Composition of the forest understory for a 100 m transect from the forest edge to its interior at each of the eight field sites. All individuals greater than 50 cm in height were counted and identified (excepting canopy trees). Here we show percentages of individuals by plant family for the three most commonly encountered families at each site ("Other" indicates the remainder of the 63 families). Composition was determined for five 5 m × 2 m plots along each transect. Of these dominant families, only Lauraceae, Melastomataceae, Piperaceae and Rubiaceae were observed at every site.

**Table S1:** Latitude and longitude of the eight sites used in this study along with the types of data recorded at each.

| Site | Latitude | Longitude | Leaves | Air | Seedling Survey |
| --- | --- | --- | --- | --- | --- |
| AJ | 8.924 N | 82.935 W | x | x |  |
| AN | 8.906 N | 82.901 W | x | x | x |
| DV | 8.959 N | 82.918 W | x | x |  |
| HB | 8.911 N | 82.907 W | x | x |  |
| LZ | 8.973 N | 82.927 W | x | x | x |
| EP | 8.973 N | 82.809 W | x | x |  |
| RB | 8.845 N | 82.901 W | x | x | x |
| RN | 8.886 N | 82.851 W | x | x | x |
| VP | 8.940 N | 82.890 W | x | x |  |

**Table S2** Abundance and diversity for Rubiaceae and non-Rubiaceae in the forest understory for our eight field sites in Coto Brus, Puntarenas, Costa Rica. Individual plants ≥ 50 cm in height in the understory were counted and identified to species in five 5 m × 2 m plots along the 100 m transect at each site (at the EP site, one plot was interrupted by a treefall, so we sampled a 3 m × 2 m and a 2 m × 2 m plot instead).

|  |  | Plant density (# per 10 m^-2^) | | Plant Species Richness | |
| --- | --- | --- | --- | --- | --- |
| Site ID | Understory plants in the family Rubiaceae (%) | Rubiaceae | Non-Rubiaceae | Rubiaceae | Total |
| AJ | 18 | 2.8 | 12.6 | 5 | 31 |
| AN | 17 | 3.0 | 14.2 | 4 | 39 |
| DV | 16 | 2.6 | 13.4 | 7 | 39 |
| EP | 37 | 6.0 | 10.4 | 3 | 35 |
| LZ | 20 | 2.8 | 11.4 | 3 | 34 |
| RB | 11 | 2.4 | 18.6 | 10 | 53 |
| RN | 2 | 0.4 | 15.8 | 3 | 31 |
| VP | 9 | 1.6 | 15.6 | 8 | 45 |

**Table S3.** Sampling effort for assessment of pathogen damage and characterization of fungal communities in leaves of 27 host plant species ranked by their phylogenetic relatedness to coffee (*Coffea arabica* L.). at each of the eight field sites (each two-letter abbreviation denotes a site). A single leaf was sampled from each plant, with 1-5 plants sampled for how many species? at each of four distances (10, 25, 50 m and 100 m) from the forest edge, except at RN in coffee, where the narrowness of the coffee field did not allow sampling at 100 m). DNA extracts from the same host species within a distance at a site were pooled prior to molecular characterization of fungal communities, Phylogenetic distance from coffee (Pdist) was determined using GenBank^®^ accessions spanning the 18S, ITS1, 5.8S, ITS2 and 26S regions of DNA. For plant species lacking sequence data, the nearest relative (indicated in parentheses) with relevant available data was substituted. *Virola sebifera* leaves inhibited DNA amplification, resulting in no viable molecular samples. Fungal communities were characterized via ITS2 metabarcoding using the fungal-specific primers ITS4^2^ and 5.8SR^3^.

| Plant family | Plant species | Pooled DNA samples | Number of leaves sampled per site | | | | | | | | Pdist | | Accession # |
| --- | --- | --- | --- | --- | --- | --- | --- | --- | --- | --- | --- | --- | --- |
|  |  |  | AJ | AN | DV | EP | LR | RB | RN | VP |  |  | |
| Rubiaceae | *Coffea arabica* L. | 30 | 16 | 16 | 20 | 16 | 16 | 15 | 16 | 16 | 0 | KP776613.1 | |
|  | *Psychotria limonensis* K.Krause | 2 | - | 4 | - | - | - | 4 | - | - | 0.354 | AF072052.1 | |
|  | *Palicourea lasiorrhachis* Oerst. | 1 | - | - | - | 1 | - | - | - | - | 0.372 | AF072009.1 | |
|  | *Palicourea aurantiibractea* (C.M.Taylor) Borhidi (*Pa. lasiorrhachis*) | 7 | - | - | 2 | - | 4 | - | - | 10 | 0.372 | AF072009.1 | |
|  | *Palicourea hebeclada* (DC.) Borhidi | 8 | - | - | 9 | - | - | 12 | 8 | 1 | 0.373 | AF071997.1 | |
|  | *Notopleura uliginosa* (Sw.) Bremek. | 8 | - | - | - | - | 11 | 8 | - | 3 | 0.384 | AF071991.1 | |
|  | *Palicourea tomentosa* (Aubl.) Borhidi | 24 | 12 | 12 | 18 | - | 9 | 12 | 16 | 20 | 0.391 | AF071993.1 | |
|  | *Psychotria marginata* Sw. | 7 | - | 1 | - | 16 | - | 17 | - | - | 0.393 | AF072049.1 | |
|  | *Rudgea cornifolia* (Kunth) Standl. (*R. stipulacea* (DC.) Steyerm.) | 8 | - | - | - | 12 | - | 8 | 12 | - | 0.394 | JX155099.1 | |
|  | *Palicourea elata* (Sw.) Borhidi | 22 | 16 | 16 | 20 | 3 | 16 | - | 4 | 20 | 0.394 | KC480541.1 | |
|  | *Psychotria panamensis* Standl. | 9 | 5 | 17 | - | 4 | - | - | 5 | - | 0.397 | FJ208641.1 | |
|  | *Palicourea deflexa* (DC.) Borhidi | 2 | - | - | 9 | - | - | - | - | - | 0.401 | AF149377.1 | |
|  | *Palicourea pilosa* (Ruiz & Pav.) Borhidi | 7 | 12 | - | - | - | 8 | - | - | 9 | 0.401 | AF149398.1 | |
|  | *Palicourea berteroana* (DC.) Borhidi | 4 | - | - | - | 16 | - | - | - | - | 0.406 | AF072003.1 | |
|  | *Palicourea longiinvolucrata* A.C.Berger (*Pa. ostreophora* (Wernham) Borhidi) | 1 | 1 | - | - | - | - | - | - | - | 0.413 | KJ804930.1 | |
|  | *Palicourea carruthersii* (Kuntze) C.M.Taylor | 9 | 5 | - | 6 | - | 6 | - | - | 2 | 0.423 | KC480544.1 | |
|  | *Palicourea brachiata* (Sw.) Borhidi | 3 | 2 | - | - | - | - | 8 | - | - | 0.448 | AF072001.1 | |
| Fagaceae | *Quercus insignis* M.Martens & Galeotti | 2 | - | - | - | - | 8 | - | - | - | 0.464 | AY040487.1 | |
| Primulaceae | *Ardisia opegrapha* Oerst. | 28 | 16 | 17 | 20 | 16 | 12 | 15 | 16 | 19 | 0.467 | JF416239.1 | |
| Sapindaceae | *Paullinia bracteosa* Radlk. | 15 | 4 | 5 | 14 | 16 | - | 12 | 16 | - | 0.520 | MZ892285.1 | |
| Burseraceae | *Protium ravenii* D.M.Porter | 1 | - | - | - | - | - | 4 | - | - | 0.522 | KJ503448.1 | |
| Lauraceae | *Ocotea atirrensis* Mez & Donn.Sm. | 4 | - | - | - | 16 | - | - | - | - | 0.532 | MF110071.1 | |
| Melastomataceae | *Miconia nutans* Donn.Sm*.* | 1 | - | - | - | - | 4 | - | - | - | 0.555 | KF821665.1 | |
| Myristicaceae | *Otoba novogranatensis* Moldenke (*Horsfieldia kingii* (Hook.f.) Warb.) | 4 | - | - | - | - | 11 | 3 | - | - | 0.616 | MW044396.1 | |
| Melastomataceae | *Miconia punctata* (Desr.) D.Don ex DC. | 14 | 12 | 14 | - | - | 8 | 12 | - | 20 | 0.620 | EU055821.1 | |
| Myristicaceae | *Virola sebifera* Aubl*.* | - | 12 | - | 5 | - | 4 | - | - | 20 | - | - | |

**Table S4**. The of number OTUs and number of reads for the 25 most abundant fungal orders in leaves and air samplers.

| Coffee Leaves | | | Forest Leaves | | | | Airborne samplers | | | |  |
| --- | --- | --- | --- | --- | --- | --- | --- | --- | --- | --- | --- |
| Order | OTUs | total reads | | Order | OTUs | total reads | | Order | OTUs | total reads | |
| unidentified | 1662 | 321706 | | unidentified | 3521 | 2400215 | | unidentified | 1024 | 629389 | |
| Pleosporales | 580 | 93765 | | Pleosporales | 932 | 621974 | | Hypocreales | 641 | 330989 | |
| Glomerellales | 83 | 35932 | | Chaetothyriales | 788 | 480099 | | Polyporales | 579 | 277062 | |
| Chaetothyriales | 385 | 25556 | | Tremellales | 208 | 228719 | | Agaricales | 921 | 186967 | |
| Agaricales | 297 | 19884 | | Hypocreales | 737 | 220091 | | Saccharomycetales | 90 | 184312 | |
| Erythrobasidiales | 26 | 15528 | | Agaricales | 657 | 204215 | | Pleosporales | 542 | 126177 | |
| Hypocreales | 295 | 12484 | | Glomerellales | 145 | 179245 | | Tremellales | 102 | 109283 | |
| Tremellales | 110 | 11034 | | Xylariales | 476 | 142047 | | Hymenochaetales | 301 | 100447 | |
| Mycosphaerellales | 56 | 11003 | | Trechisporales | 279 | 117626 | | Eurotiales | 76 | 99195 | |
| Capnodiales | 54 | 9356 | | Diaporthales | 123 | 110360 | | Trechisporales | 295 | 56627 | |
| Xylariales | 248 | 9346 | | Amphisphaeriales | 27 | 80689 | | Glomerellales | 70 | 46995 | |
| Amphisphaeriales | 22 | 5571 | | Orbiliales | 119 | 60901 | | Chaetothyriales | 310 | 38248 | |
| Diaporthales | 67 | 4302 | | Mycosphaerellales | 95 | 57595 | | Russulales | 146 | 28896 | |
| Sordariales | 34 | 2790 | | Helotiales | 128 | 46704 | | Auriculariales | 188 | 25559 | |
| Spiculogloeales | 6 | 2194 | | Capnodiales | 174 | 40511 | | Xylariales | 287 | 24623 | |
| Trechisporales | 125 | 2169 | | Exobasidiales | 12 | 30786 | | Coniochaetales | 7 | 23627 | |
| Phomatosporales | 12 | 1941 | | Polyporales | 185 | 24533 | | Mycosphaerellales | 65 | 21963 | |
| Trichosphaeriales | 19 | 1706 | | Eurotiales | 50 | 23229 | | Trichosporonales | 11 | 21690 | |
| Helotiales | 57 | 1565 | | Cantharellales | 103 | 22544 | | Exobasidiales | 17 | 12076 | |
| Dothideomycetes *incertae sedis* | 5 | 1499 | | Hymenochaetales | 121 | 21413 | | Amphisphaeriales | 17 | 10121 | |
| Polyporales | 109 | 1460 | | Lecanorales | 84 | 17830 | | Sordariales | 48 | 8374 | |
| Cantharellales | 48 | 1250 | | Agaricostilbales | 39 | 16905 | | Sporidiobolales | 19 | 8130 | |
| Orbiliales | 33 | 1097 | | Saccharomycetales | 21 | 16887 | | Diaporthales | 72 | 7833 | |
| Hymenochaetales | 63 | 751 | | Botryosphaeriales | 25 | 15889 | | Capnodiales | 74 | 7398 | |
| Venturiales | 18 | 654 | | Cystofilobasidiales | 3 | 12054 | | Cantharellales | 97 | 6771 | |

**Figure S3.** Relative abundance (**A**) and number of OTUs (**B**) on coffee leaves of the ten most abundant fungal orders for which a taxonomic designation could be made. Reads assigned to Pleosporales had nearly three times higher relative abundance than the next most abundant order, and they exhibited the greatest richness, making the order a strong candidate coffee-associated group to assess for spillover into adjacent forest.

**Table S5.** Taxa captured in air samples that were significantly more abundant in forest samplers the more closely related they were to coffee according to our GJAM model^4^ (Fig. 4). The ‘lowest taxon’ indicates the lowest available taxonomic designation for each OTU when no closest-matching genus was identified.

| OTU ID | Effect Estimate ± SE | | | Order | Genus/lowest taxon |
| --- | --- | --- | --- | --- | --- |
| OTU63751 | -0.834 | **±** | 0.10 | Tremellales | unidentified |
| OTU41763 | -0.565 | **±** | 0.11 | Hypocreales | unidentified |
| OTU17663 | -0.377 | **±** | 0.10 | unidentified | unidentified |
| OTU51256 | -0.369 | **±** | 0.11 | unidentified | unidentified |
| OTU16868 | -0.358 | **±** | 0.10 | Agaricales | unidentified |
| OTU15029 | -0.328 | **±** | 0.11 | Cystobasidiales | *Occultifur* |
| OTU49031 | -0.326 | **±** | 0.10 | Pleosporales | *Periconia* |
| OTU67068 | -0.323 | **±** | 0.11 | Tremellales | *Hannaella* |
| OTU65542 | -0.322 | **±** | 0.11 | Tremellales | *Vishniacozyma* |
| OTU57830 | -0.316 | **±** | 0.11 | Pleosporales | *Neosetophoma* |
| OTU47862 | -0.314 | **±** | 0.11 | Pleosporales | *Alternaria* |
| OTU53698 | -0.311 | **±** | 0.10 | Hypocreales | unidentified |
| OTU9668 | -0.307 | **±** | 0.10 | Agaricales | *Coprinellus* |
| OTU6549 | -0.306 | **±** | 0.11 | Ustilaginales | *Moesziomyces* |
| OTU53298 | -0.296 | **±** | 0.10 | Pleosporales | *Paraphaeosphaeria* |
| OTU39973 | -0.295 | **±** | 0.11 | Eurotiales | *Penicillium* |
| OTU54961 | -0.284 | **±** | 0.11 | Pleosporales | Didymosphaeriaceae |
| OTU5538 | -0.279 | **±** | 0.08 | Hypocreales | unidentified |
| OTU16786 | -0.279 | **±** | 0.11 | Agaricales | *Ripartitella* |
| OTU10059 | -0.258 | **±** | 0.11 | Exobasidiales | *Meira* |
| OTU29536 | -0.255 | **±** | 0.12 | Hypocreales | *Simplicillium* |
| OTU51376 | -0.252 | **±** | 0.10 | Pleosporales | unidentified |
| OTU58014 | -0.241 | **±** | 0.10 | Capnodiales | *Cladosporium* |
| OTU25405 | -0.226 | **±** | 0.09 | Hypocreales | *Thelonectria* |
| OTU51503 | -0.225 | **±** | 0.11 | Pleosporales | *Periconia* |
| OTU41042 | -0.223 | **±** | 0.08 | Chaetothyriales | *Cyphellophora* |
| OTU47506 | -0.220 | **±** | 0.11 | Eurotiales | *Penicillium* |
| OTU57907 | -0.220 | **±** | 0.10 | unidentified | Cladosporiaceae. |
| OTU18761 | -0.215 | **±** | 0.09 | Agaricales | *Coprinopsis* |
| OTU52630 | -0.211 | **±** | 0.10 | Pleosporales | Phaeosphaeriaceae |
| OTU60564 | -0.190 | **±** | 0.05 | unidentified | unidentified |
| OTU32517 | -0.183 | **±** | 0.09 | unidentified | unidentified |
| OTU46995 | -0.152 | **±** | 0.07 | Hypocreales | *Ovicillium* |

**Table S6.** Taxa of foliar fungi that that were significantly more abundant in forest samplers the closer they were to the coffee forest border according to our GJAM model^4^ (Fig. 8). The ‘lowest taxon’ indicates the lowest available taxonomic designation for each OTU when a closest matching genus was not identified.

| OTU ID |  | Effect Estimate ± SE | | | | Order | | Genus/lowest taxon | |
| --- | --- | --- | --- | --- | --- | --- | --- | --- | --- |
| OTU26796 | -0.368 | | **±** | 0.17 | Chaetothyriales | | *Cyphellophora* | |  |
| OTU58291 | -0.342 | | **±** | 0.15 | Sordariales | | Chaetomiaceae | |  |
| OTU50968 | -0.303 | | **±** | 0.12 | Pleosporales | | Pleosporales | |  |
| OTU60839 | -0.289 | | **±** | 0.11 | Mycosphaerellales | | *Pseudocercospora* | |  |
| OTU22895 | -0.282 | | **±** | 0.15 | Orbiliales | | *Lecophagus* | |  |
| OTU40557 | -0.275 | | **±** | 0.10 | unidentified | | unidentified | |  |
| OTU44774 | -0.273 | | **±** | 0.14 | unidentified | | unidentified | |  |
| OTU53372 | -0.268 | | **±** | 0.13 | Pleosporales | | *Setophoma* | |  |
| OTU57332 | -0.264 | | **±** | 0.15 | Helotiales | | Helotiales | |  |
| OTU53893 | -0.255 | | **±** | 0.10 | Pleosporales | | Pleosporales | |  |
| OTU39067 | -0.255 | | **±** | 0.10 | Amphisphaeriales | | *Pestalotiopsis* | |  |
| OTU53232 | -0.249 | | **±** | 0.07 | Pleosporales | | Didymellaceae | |  |
| OTU22070 | -0.225 | | **±** | 0.11 | Orbiliales | | Orbiliales | |  |
| OTU52567 | -0.209 | | **±** | 0.10 | Sordariales | | Sordariales | |  |
| OTU9668 | -0.204 | | **±** | 0.07 | Agaricales | | *Coprinellus* | |  |
| OTU32679 | -0.188 | | **±** | 0.10 | Tremellales | | *Papiliotrema* | |  |
| OTU55326 | -0.187 | | **±** | 0.07 | Pleosporales | | *Setophoma* | |  |
| OTU41870 | -0.185 | | **±** | 0.10 | Amphisphaeriales | | *Pestalotiopsis* | |  |
| OTU56681 | -0.153 | | **±** | 0.08 | unidentified | | Lecanoromycetes | |  |

**Table S7.** Taxa among the 1,000 most abundant OTUs in the coffee that were significantly more abundant on forest plants the more closely related they were to coffee, according to our GJAM^4^ model^4^ (Fig 8). The ‘lowest taxon’ indicates the lowest available taxonomic designation for each OTU when a closest matching genus was not identified.

| otu_id | Effect Estimate ± SE | | | Order | Genus/lowest taxon |  |
| --- | --- | --- | --- | --- | --- | --- |
| OTU51335 | -0.569 | **±** | 0.16 | Glomerellales | *Colletotricum* |  |
| OTU48382 | -0.501 | **±** | 0.07 | Glomerellales | *Colletotrichum* |  |
| OTU29712 | -0.451 | **±** | 0.17 | Chaetothyriales | Trichomeriaceae |  |
| OTU48023 | -0.415 | **±** | 0.14 | Pleosporales | *Galiicola* |  |
| OTU39306 | -0.412 | **±** | 0.18 | Chaetothyriales | *Strelitziana* |  |
| OTU24069 | -0.404 | **±** | 0.15 | Chaetothyriales | Herpotrichiellaceae |  |
| OTU47101 | -0.400 | **±** | 0.15 | Chaetothyriales | *Cladophialophora* |  |
| OTU24159 | -0.389 | **±** | 0.17 | Hypocreales | *Xepicula* |  |
| OTU41027 | -0.383 | **±** | 0.14 | unidentified | unidentified |  |
| OTU50252 | -0.368 | **±** | 0.14 | Glomerellales | *Colletotrichum* |  |
| OTU38480 | -0.364 | **±** | 0.17 | Chaetothyriales | Cyphellophoraceae |  |
| OTU61431 | -0.349 | **±** | 0.15 | Mycosphaerellales | *Cercospora* |  |
| OTU44049 | -0.347 | **±** | 0.13 | Pleosporales | *Spegazzinia* |  |
| OTU33529 | -0.344 | **±** | 0.14 | unidentified | Sordariomycetes |  |
| OTU9523 | -0.341 | **±** | 0.12 | Agaricales | *Crepidotus* sp. |  |
| OTU44098 | -0.330 | **±** | 0.15 | unidentified | unidentified |  |
| OTU55212 | -0.324 | **±** | 0.11 | Pleosporales | *Setophoma* |  |
| OTU28589 | -0.322 | **±** | 0.15 | Orbiliales | *Dactylella* |  |
| OTU56681 | -0.322 | **±** | 0.08 | unidentified | Lecanoromycetes |  |
| OTU32178 | -0.319 | **±** | 0.14 | unidentified | Sordariomycetes |  |
| OTU50449 | -0.317 | **±** | 0.17 | Pleosporales | *Setophoma* |  |
| OTU48016 | -0.315 | **±** | 0.10 | Venturiales | Sympoventuriaceae |  |
| OTU36783 | -0.313 | **±** | 0.12 | Chaetothyriales | *Strelitziana* |  |
| OTU53232 | -0.305 | **±** | 0.07 | Pleosporales | Didymellaceae |  |
| OTU49862 | -0.301 | **±** | 0.14 | Pleosporales | Didymellaceae |  |
| OTU66135 | -0.301 | **±** | 0.14 | unidentified | Bulleribasidiaceae |  |
| OTU37043 | -0.300 | **±** | 0.11 | Chaetothyriales | *Strelitziana* |  |
| OTU15725 | -0.296 | **±** | 0.13 | Agaricales | *Arachnion* |  |
| OTU33504 | -0.295 | **±** | 0.12 | unidentified | unidentified |  |
| OTU58151 | -0.293 | **±** | 0.11 | Pleosporales | *Neosetophoma* |  |
| OTU55256 | -0.291 | **±** | 0.11 | unidentified | unidentified |  |
| OTU10838 | -0.290 | **±** | 0.12 | Erythrobasidiales | *Erythrobasidium* |  |
| OTU53194 | -0.289 | **±** | 0.12 | Sordariales | Podosporaceae |  |
| OTU16958 | -0.288 | **±** | 0.12 | unidentified | unidentified | |
| OTU47862 | -0.287 | **±** | 0.12 | Pleosporales | *Alternaria* | |
| OTU26787 | -0.287 | **±** | 0.14 | unidentified | unidentified | |
| OTU54978 | -0.286 | **±** | 0.11 | Xylariales | Bartaliniaceae | |
| OTU20521 | -0.285 | **±** | 0.10 | Orbiliales | Orbiliales | |
| OTU54126 | -0.284 | **±** | 0.08 | Pleosporales | Pleosporales | |
| OTU48011 | -0.283 | **±** | 0.12 | Amphisphaeriales | Pestalotiopsidaceae. | |
| OTU39590 | -0.282 | **±** | 0.11 | unidentified | Strelitzianaceae | |
| OTU67200 | -0.279 | **±** | 0.10 | unidentified | unidentified | |
| OTU13313 | -0.278 | **±** | 0.12 | Agaricales | *Psilocybe* | |
| OTU57184 | -0.275 | **±** | 0.07 | Pleosporales | *Phaeosphaeria* | |
| OTU34633 | -0.275 | **±** | 0.11 | Chaetothyriales | Trichomeriaceae | |
| OTU11831 | -0.274 | **±** | 0.13 | unidentified | Tremellomycetes | |
| OTU57306 | -0.270 | **±** | 0.10 | Pleosporales | Phaeosphaeriaceae | |
| OTU52827 | -0.261 | **±** | 0.13 | Pleosporales | Pleosporales | |
| OTU54100 | -0.259 | **±** | 0.14 | Xylariales | Xylariales | |
| OTU66654 | -0.254 | **±** | 0.11 | unidentified | unidentified | |
| OTU15695 | -0.254 | **±** | 0.12 | Agaricales | *Hypholoma brunneum* | |
| OTU52841 | -0.252 | **±** | 0.12 | Xylariales | Apiosporaceae | |
| OTU53951 | -0.252 | **±** | 0.11 | unidentified | unidentified | |
| OTU29409 | -0.251 | **±** | 0.12 | Hypocreales | unidentified | |
| OTU25892 | -0.245 | **±** | 0.12 | unidentified | unidentified | |
| OTU31221 | -0.244 | **±** | 0.09 | Chaetothyriales | Trichomeriaceae | |
| OTU58722 | -0.243 | **±** | 0.09 | unidentified | Lecanoromycetes | |
| OTU61826 | -0.243 | **±** | 0.11 | unidentified | unidentified | |
| OTU26149 | -0.242 | **±** | 0.08 | Xylariales | *Fusidium* | |
| OTU67650 | -0.241 | **±** | 0.12 | unidentified | unidentified | |
| OTU28356 | -0.24 | **±** | 0.10 | unidentified | Trichomeriaceae | |
| OTU25135 | -0.233 | **±** | 0.11 | Tremellales | *Papiliotrema* | |
| OTU67403 | -0.230 | **±** | 0.09 | Tremellales | *Bulleribasidium* | |
| OTU42888 | -0.226 | **±** | 0.10 | Chaetothyriales | Trichomeriaceae | |
| OTU57217 | -0.225 | **±** | 0.08 | Pleosporales | Pleosporales | |
| OTU34593 | -0.224 | **±** | 0.11 | unidentified | Trichomeriaceae | |
| OTU18201 | -0.223 | **±** | 0.11 | Agaricales | *Psathyrella* | |
| OTU52567 | -0.222 | **±** | 0.10 | Sordariales | Sordariales | |
| OTU37689 | -0.221 | **±** | 0.11 | Chaetothyriales | Chaetothyriaceae | |
| OTU49734 | -0.219 | **±** | 0.09 | unidentified | Didymellaceae | |
| OTU31514 | -0.218 | **±** | 0.10 | Chaetothyriales | Chaetothyriales | |
| OTU53670 | -0.215 | **±** | 0.10 | Trichosphaeriales | *Nigrospora* | |
| OTU67095 | -0.21 | **±** | 0.11 | Tremellales | *Hannaella* | |
| OTU38294 | -0.206 | **±** | 0.09 | unidentified | unidentified | |
| OTU47997 | -0.205 | **±** | 0.09 | Pleosporales | unidentified | |
| OTU28297 | -0.203 | **±** | 0.10 | Tremellales | *Trimorphomyces* | |
| OTU67241 | -0.202 | **±** | 0.08 | unidentified | unidentified | |
| OTU53802 | -0.201 | **±** | 0.09 | Pleosporales | *Paraconiothyrium* | |
| OTU51250 | -0.192 | **±** | 0.10 | unidentified | unidentified | |
| OTU41050 | -0.191 | **±** | 0.09 | unidentified | Dothideomycetes | |
| OTU58024 | -0.190 | **±** | 0.09 | Capnodiales | *Cladosporium* | |
| OTU34069 | -0.185 | **±** | 0.05 | Glomerellales | *Plectosphaerella* | |
| OTU47014 | -0.182 | **±** | 0.10 | unidentified | unidentified | |
| OTU60016 | -0.181 | **±** | 0.10 | Pleosporales | *Leptospora* | |
| OTU47966 | -0.179 | **±** | 0.09 | Diaporthales | Diaporthaceae | |
| OTU34318 | -0.17 | **±** | 0.08 | Chaetothyriales | Trichomeriaceae | |
| OTU67154 | -0.163 | **±** | 0.08 | Tremellales | *Hannaella* | |
| OTU25868 | -0.157 | **±** | 0.06 | Xylariales | *Fusidium* | |
| OTU9668 | -0.155 | **±** | 0.07 | Agaricales | *Coprinellus*. | |
| OTU44265 | -0.152 | **±** | 0.08 | unidentified | Phyllostictaceae | |
| OTU60045 | -0.152 | **±** | 0.07 | Helotiales | *Tetracladium* | |
| OTU21838 | -0.148 | **±** | 0.08 | Agaricales | Strophariaceae | |

| Dataset | Model # | Model Type | Data included | Response Variable | Fixed Effect | Effect Estimate | p value |
| --- | --- | --- | --- | --- | --- | --- | --- |
| Leaf images | 1 | Binomial GLMM | All | leafspot incidence | Vegetation type | -2.43 | 0.0001 |
|  | 2 | Binomial GLMM | Forest Rubiaceae | leafspot incidence | pdist | -0.12 | 0.6652 |
|  |  |  |  |  | dist | -0.57 | 0.0367 |
|  |  |  |  |  | pdist*dist | 0.18 | 0.4724 |
|  | 3 | Binomial GLMM | Forest non-Rubiaceae | leafspot incidence | pdist | 0.51 | 0.1226 |
|  |  |  |  |  | dist | -0.06 | 0.7826 |
|  |  |  |  |  | pdist*dist | -0.22 | 0.3355 |
| Seedling survey | 4 | LMER | All | Pathogen damage | pdist | 0.35 | 0.0263 |
|  |  |  |  |  | dist | -0.36 | 0.0142 |
|  |  |  |  |  | pdist*dist | 0.52 | 0.0148 |
| Fungal DNA from air samplers | 5 | Negative Binomial GLMM | All | total reads | dist | 0.005 | 0.0236 |
|  | 6 | Beta Binomial GLMM | All | RA of Pleosporales | dist | -0.004 | 1.28 × 10⁻⁷ |
|  | 7 |  | Forest Interior (10m-100m) | RA of Pleosporales | dist | -0.01 | 0.0272 |
| Fungal DNA from leaves | 8 |  | All leaves | RA of Pleosporales | dist | -0.01 | 0.0001 |
|  | 9 |  | Forest Rubiaceae | RA of Pleosporales | dist | -0.004 | 0.0007 |
|  | 10 |  | Forest non-Rubiaceae | RA of Pleosporales | dist | -0.001 | 0.4928 |

**Table S8:** Summary of non-GJAM linear models used in this study**.** The term “dist” refer to physical distance along the transect and “pdist” refers to phylogenetic distance from coffee. “dist*pdist” refers to the interaction of these two terms. RA = relative abundance.

A complete table of all model statistics is available with the source data.

**Figure S4** (**A**) Coverage-standardized richness (q = 0) and (**B**) coverage-standardized diversity (q = 1, Shannon-order Hill diversity) of foliar fungal communities across coffee leaves, forest Rubiaceae, and forest non-Rubiaceae hosts. Points represent individual leaf samples, boxplots show medians and interquartile ranges, and violins indicate distribution density. Both richness and diversity differed significantly among host groups (Kruskal–Wallis tests, p = 1.10 × 10⁻¹⁴; 4.62 × 10⁻¹⁰, or both metrics). Coffee leaves harbored the lowest diversity, forest Rubiaceae the highest, and forest non-Rubiaceae intermediate values (pairwise Wilcoxon tests with BH correction, all P ≤ 0.001; exact *p* values provided in the Results) (**C**) Three-dimensional NMDS ordination of Hellinger-transformed fungal community composition based on Bray-Curtis distances (Stress = 0.173). Points represent pooled leaf communities of a sampled species at a given sampling distance at a given site colored by host type; shaded ellipses are descriptive visualizations of within-group ordination spread and overlap across NMDS projections. Community composition differed significantly among host types. In forest-only samples, composition also differed between Rubiaceae and non-Rubiaceae hosts (F₃,₁₈₇ = 1.74, R² = 0.027, p = 0.001). Fungal communities of forest non-Rubiaceae leaves were more heterogeneous than Forest Rubiaceae communities (PERMDISP: *F*₁,₁₈₉ = 29.57, *P* < 0.001). Here, *n* = 221 (coffee = 30, forest Rubiaceae = 122, forest non-Rubiaceae = 69).

**Table S9.** Fungal-specific forward and reverse ITS2 primer sequences with Nextera adapters used for metabarcoding of leaves and airborne samples

| Primer | Direction | Target Region | Sequence |
| --- | --- | --- | --- |
| ITS4_Nextera | Forward | fungalITS2 | GTCTCGTGGGCTCGGAGATGTGTATAAGAGACAGTCCTCCGCTTATTGATATGC |
| 5.8SR_Nextera | Reverse | fungalITS2 | TCGTCGGCAGCGTCAGATGTGTATAAGAGACAGTCGATGAAGAACGCAGCG |

**Supplementary References**

1.	Goossens, D. & Offer, Z. Y. Wind tunnel and field calibration of six aeolian dust samplers. *Atmos. Environ.* **34**, 1043–1057 (2000).

2.	White, T. J., Bruns, T. & Lee, S. Analysis of phylogenetic relationships by amplification and direct sequencing of ribosomal RNA genes. *Guide Methods Appl.* (1990).

3.	Vilgalys, R. & Hester, M. Rapid genetic identification and mapping of enzymatically amplified ribosomal DNA from several Cryptococcus species. *J. Bacteriol.* **172**, 4238–4246 (1990).

4.	Clark, J. S., Nemergut, D., Seyednasrollah, B., Turner, P. J. & Zhang, S. Generalized joint attribute modeling for biodiversity analysis: median-zero, multivariate, multifarious data. *Ecol. Monogr.* **87**, 34–56 (2017).
